# A Pan-H5N1 Multiepitope DNA Vaccine Construct Targeting Some Key Proteins of the Clade 2.3.4.4b Using AI-Assisted Epitope Mapping and Molecular Docking

**DOI:** 10.3390/v17091152

**Published:** 2025-08-22

**Authors:** Nithyadevi Duraisamy, Abid Ullah Shah, Mohd Yasir Khan, Mohammed Cherkaoui, Maged Gomaa Hemida

**Affiliations:** 1 Department of Computer Science, College of Digital Engineering and Artificial Intelligence, Long Island University, Brooklyn, NY 11201, USA; nithyadevi.duraisamy@liu.edu (N.D.); mohd.yasirkhan@liu.edu (M.Y.K.); mohammed.cherkaoui@liu.edu (M.C.); 2 Department of Veterinary Biomedical Sciences, College of Veterinary Medicine, Long Island University, 720 Northern Boulevard, Brookville, NY 11548, USA; abidullah.shah@liu.edu

**Keywords:** highly pathogenic avian influenza virus, H5N1, clade 2.3.4.4b, epitope mapping, DNA vaccine, in silico prediction, molecular docking, IL8, TL3, TLR7

## Abstract

The presently used vaccines do not offer solid immunity/protection against the currently circulating strains of the H5N1 viruses. We aim to design a pan-H5N1 vaccine that protects birds against the presently circulating clade 2.3.4.4b in chickens. We used AI tools, including epitope mapping, molecular docking, and immune simulation, to design a multiepitope DNA vaccine including the top-ranked B and T cell epitopes within four major proteins (HA, NA, NP, and M2) of H5N1 clade 2.3.4.4b. We selected the top-ranked 12 epitopes and linked them together using linkers. The designed vaccine is linked to IL-18 as an adjuvant. The molecular docking results showed a high binding affinity of those predicted epitopes from the MHC I and MHC II classes of molecules with chicken alleles. The immune simulation results showed that the designed vaccine has the potential to stimulate the host immune response, including antibody and cell-mediated immunity in chickens and other birds. We believe this vaccine is going to be a universal vaccine that offers good protection against HPAI-H5N1 clade 2.3.4.4b. We are reporting the successful molecular cloning of a recombinant multiepitope-based vaccine spanning some key epitopes within some key proteins of the currently circulating H5N1 clade 2.3.4.4b. These designed vaccines could be a great positive impact on the protection of birds and various species of animals, as well as humans, against the HP-H5N1 influenza virus. Further studies are required to validate this vaccine candidate in chickens.

## 1. Introduction

The highly pathogenic avian influenza H5N1 (HPAIV) virus continues to pose a significant risk to the poultry industry. There is also a risk of spillover to humans, which results in the death of some affected patients [1,2,3]. There is a continuous active dynamic change in the virus’s genetic material for many reasons, including the poor proofreading capability of the viral polymerase enzyme, the antigenic shift/drift, the possibility of reassortment, and recombination [4,5]. This pattern of frequent changes in the viral genomes resulted not only in the emergence of new viruses or clades of the same lineage of the virus but also could hamper the success of the currently used vaccine and diagnostic assays for the HPAIV. There is a mandate for the active monitoring of these viruses at the genomic level to monitor the emergence of new viruses that might have any abnormal genotypic/phenotypic patterns of the virus. There is also a high demand for the generation of novel diagnostic assays and vaccines that could detect/protect against the currently circulating strains of the virus in the field. Influenza virus type A (IAV) has segmented genomes consisting of 7–8 segments. Each segment of the viral genome encodes at least one important protein. Influenza viruses have several important proteins, including the Hemagglutinin (HA), the neuraminidase (NA), the nucleoprotein (NP), and the matrix protein (M). This is in addition to some nonstructural proteins (NSP1 and NSP2, in addition to the viral polymerase, which consists of three subunits called PA, PB2, and PB2 proteins) [6]. The AIV is classified based on its HA and NA proteins into 19 HA and 11 NA subtypes, respectively [6]. The HA, NA, and M proteins are expressed on the surface of viral particles. The NP wraps the viral genome to form the viral nucleoprotein [6]. The HA epitopes proved to trigger high neutralizing antibodies in the infected/vaccinated host [7]. The HA sequence is prone to frequent changes driven by antigenic shift and drift, which enable the virus to evade the host immune response [7]. The NA protein plays several key roles in influenza virus replication, pathogenesis, and immune evasion as well [7]. The AIV-NP also plays an important role in the suppression of the host immune response through the activation of the mitophagy pathways in the infected cells [8]. The matrix protein of the IVA consists of M1 and M2 proteins. Both proteins play important roles in the immune response/evasion against the IVA in the host. The M2 protein plays an essential role in viral immune evasion by modulating the autophagy pathways in the infected cell through the prevention of the fusion between the autophagosome and the lysosome, which augments the viral immune evasion strategies [9]. There are several approaches for the preparation of AIV vaccines, including live attenuated, inactivated, recombinant, and DNA vaccines. Each type of vaccine has advantages and disadvantages. There is an ongoing trend of using AI tools in vaccine design and development for many viral diseases in humans, animals, and birds [10,11,12,13]. The application of AI tools accelerates epitope prediction, antigen selection, and immune response modeling, particularly in the context of emerging and re-emerging infectious diseases [10,11,12,13]. The most feasible example of the application of AI tools in vaccine design and evaluation is the development of the mRNA vaccine against SARS-CoV-2 [14].

DNA vaccines for AIV hold great promise, especially these days, for several reasons [15]. The cost of production of the DNA vaccine is very low compared to other types of vaccines, the possibility of upgrading DNA vaccines to match any changes in the viral genetic materials and in the case of an emergence compared to the other types of vaccines, the mass production of DNA vaccine could be synthesized in a remarkable short period maintaining their long term stability [15]. However, one of the major concerns of the DNA vaccines is the delivery method and their duration of action in the vaccinated hosts. Several approaches have been recently adapted to prolong the actions of the DNA vaccines and to protect them from the actions of the host DNase enzymes. Several approaches have been developed to improve the quality of the DNA vaccines and to prolong their actions, including encapsulation with various types of nanoparticles, particularly lipid nanoparticles and chitosan. The incorporation of the IVA DNA vaccine against the M protein with chitosan administered intranasally produced a prolonged immune response in mice [16]. Encapsulation of the DNA vaccine with lipid nanoparticles enhanced the immune response of vaccinated pigs against the H1N1 virus infection [17]. In the present study, we designed a multiepitope DNA-based vaccine, including the top-ranked B cell and T cell epitopes within the four major proteins (HA, NA, NP, and M2) of H5N1 clade 2.3.4.4b. The in silico immune simulation of the designed vaccine showed promising results in the induction of a robust immune response in the vaccinated birds against this clade of the AIV in birds. However, these studies require further experimental validation using these vaccines in chickens and other birds, such as turkeys.

## 2. Materials and Methods

**Figure 1:**
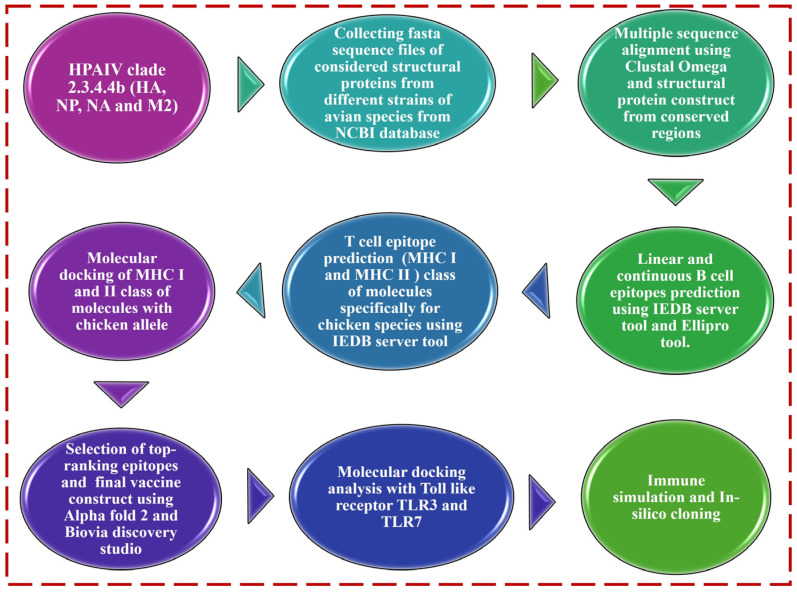
Proposed model workflow for multiepitope vaccine (MEV) construct and molecular docking analysis with Toll-like receptors (TLR3 and TLR4).

Figure 1 illustrates the steps of selection, prediction, and molecular docking analysis of structural proteins (HA, NP, NA and M2) of chicken influenza virus H5N1 clade 2.3.4.4b with chicken allele (MHC class of molecules) and Toll-like receptors (TLR3 and TLR7) through a public database (NCBI), the utilization of various AI-derived computational techniques, immune simulation, and in silico cloning.

### 2.1. Retrieval of the H5N1 Clade 2.3.4.4b Protein Sequences

A total of 279 isolate sequences belonging to H5N1 clade 2.3.4.4b, including four major viral proteins (Hemagglutinin (HA), nucleoprotein (NP), neuraminidase (NA), and the matrix protein (M2)), were retrieved from the National Center for Biotechnology (NCBI) database (https://www.ncbi.nlm.nih.gov/protein (accessed on 1 July 2025)). These sequences include 115 chickens, 40 ducks, 30 turkeys, migratory birds = 43 (including Red-tailed hawk, Peregrine falcon, American wigeon, and Backyard bird), and Canadian goose = 51. The Supplementary Excel Files (Tables S1–S4) present information about these sequences.

### 2.2. The Multiple Sequence Alignment (MSA) and Phylogenetic Analysis

The MSA for H5N1 clade 2.3.4.4b HA, M2, NA, and NP proteins was conducted independently using Geneious Prime software (V 2024.0.3) (https://www.geneious.com/ (accessed on 1 August 2025)) and the clustal Omega server tool (https://www.ebi.ac.uk/jdispatcher/msa/clustalo (accessed on 1 August 2025)). The highly conserved consensus sequences per protein showing 100% identity were further considered for the epitope mapping. The phylogenetic analysis for H5N1 HA, M2, NA, and NP was performed using the neighbor-joining tree method on Geneious Prime software. The resulting Nexus file was exported and visualized using iTOL (https://itol.embl.de/ (accessed on 1 August 2025)) [18] (Supplementary Figures S1–S4).

### 2.3. Mapping B Cell Epitopes Within the Avian H5N1 Clade 2.3.4.4b Major Proteins (HA, NA, NP, and M2)

#### 2.3.1. Prediction of the Linear B Cell Epitopes

To map the B cell epitopes from the generated consensus sequences of the four proteins (HA, NA, NP, and M2), we used the BCPREDS (BepiPred 2.0) (http://services.healthtech.dtu.dk/services/BepiPred-2.0/ (accessed on 1 August 2025)) and the IEDB analysis resource server (http://tools.iedb.org/bcell/ (accessed on 1 August 2025)) as described [19]. We adjusted the length of the target epitopes to 20 mers. The identified epitopes were further filtered based on their antigenicity, allergenicity, toxicity, and solubility, as previously described [20].

#### 2.3.2. Prediction of the Discontinuous/Conformational B Cell Epitopes

The discontinuous/conformational epitopes were predicted using the Ellipro server (http://tools.iedb.org/ellipro/ (accessed on 1 August 2025)). The parameter was set at 0.5 for the minimum score and 6 Å for the maximum distance [21]. This method is based on the protein antigen’s 3D structure, solvent accessibility, and flexibility. The Chimera software was used to display the position of predicted epitope clusters on the 3D structures of all the structural proteins.

### 2.4. Mapping of the T-Lymphocyte Epitopes Within the Avian H5N1 Clade 2.3.4.4b Major Proteins (HA, NA, NP, and M2)

#### 2.4.1. Prediction of the Cytotoxic T-Lymphocyte Epitopes (MHC Class I Molecules)

The IEDB server (http://tools.iedb.org/main/tcell (accessed on 1 August 2025)) was used to predict the cytotoxic T-lymphocytes (CTLs) and the helper T-lymphocytes epitopes that bind to MHC-I and MHC-II, respectively. The epitope binding predictor NetMHCpan 4.1 BA (version 2023.09) was used to sort the peptides by inhibitory concentration (IC50) value for the epitope prediction [22]. The source species were entered as humans with peptide lengths of 9–10 and associated human alleles (HLA-A, HLA-B, and HLA-C). The mapped epitope list was filtered according to its percentile rank and IC50 value. It was then evaluated for its antigenic, non-allergic, non-toxic, and solubility characteristics with further refinement.

#### 2.4.2. Prediction of the Helper T-Lymphocyte Epitopes (MHC Class II Molecules)

We used the IEDB analysis tool (http://tools.iedb.org/mhcii/ (accessed on 1 August 2025)) to predict the MHC class II binding molecules using NetMHCII pan 4.1 BA (recommended binding predictor: 2023.09), using the human allele (HLA-DP, HLA-DQ, and HLA-DR) as a selective species based on percentile rank and IC50 value [23]. Following refinement of their antigenic, non-allergic, non-toxic, and solubility properties, the filtered epitopes were assessed and chosen following a percentile rank score of less than 10. It was then evaluated for antigenic, non-allergic, non-toxic, and solubility characteristics with further filtration to be utilized in the designed vaccine construct. The reference sequence of each of the four proteins (HA, NP, NA, and M2) of H5N1 clade 2.3.4.4b was analyzed against the human alleles (HLA-DR, DQ, DP) using IEDB MHC-II binding prediction tools with a percentile rank of less than or equal to 10. A large number of epitopes were obtained as a result of the four proteins, and the epitopes were initially filtered based on the half minimal inhibitory concentration (IC50) and percentile rank, followed by filtering out based on allergenicity, antigenicity, non-toxicity, and solubility. Here, to predict the MHC class II molecules for the chicken alleles (Gaga BLB1 and Gaga BLB2), we used the mixmhc2pred.gfellerlab.org server tool, where a single fragment of amino acids with a length of 15 mers was provided as input and predicted the results based on percentile rank.

### 2.5. Molecular Docking and Analysis of the Binding Interaction Between the Predicted T Cell Epitopes with Chicken MHC-I and MHC-II Alleles

The 3D structures of the short-listed CTL and HTL epitopes were modeled using the PEP-FOLD3 (de novo peptide structure prediction) server (https://mobyle.rpbs.univ-paris-diderot.fr/cgi-bin/portal.py#forms::PEP-FOLD3: PEP-FOLD3: faster de novo structure prediction for linear peptides in solution and in complex | Nucleic Acids Research | Oxford Academic (accessed on 1 August 2025)), using the sOPEP energy function to cluster peptide conformation [24]. The sequences of chicken MHC alleles (BL, BF) were retrieved either from the Protein Data Bank (PDB) or the Uniprot and were generated using the Biovia Discovery Studio [25]. The molecular docking of the selected CTL and HTL epitopes with their respective MHC alleles was performed using the HADdock (http://hdock.phys.hust.edu.cn/ (accessed on 1 August 2025)) [26,27]. The binding interactions and key residue contact were visualized and further evaluated using the PDBsum server [28]. The selected epitopes were further filtered based on their docking binding affinity and confidence score and processed to design the vaccine construct.

### 2.6. Assembly of the Multiepitope Using the Top-Ranked Epitopes

The primary arrangements of the vaccine sequence were performed by fusing the B cell and T cell predicted epitopes that were filtered out based on the predicted antigenic, non-allergic, non-toxic, and good solubility of the selected epitopes. The top-ranked B and T cell epitopes were linked using KK, GPGPG, and AAY as linkers. The C-terminal ends of the vaccine construct were linked with chicken IL-18 (Accession No. CAB96214) as an adjuvant after separation with the EAAAK linker [29,30]. Moreover, the sequence was provided with a 6 × His-tag (H) attached to the C-terminus for purification and identification of the vaccine upon expression.

### 2.7. Codon Optimization and In Silico Cloning of the Multiepitope Vaccine Construct

The multiepitope vaccine was optimized and cloned into the expression vector to ensure the potential for effective cloning. Hence, the reverse translation of the vaccine protein sequences into a respective DNA sequence was performed using the Vector builder software (https://en.vectorbuilder.com/tool/codon-optimization.html (accessed on 1 August 2025)). The codon adaptive index (CAI) value and the GC content of the multiepitope construct were also calculated as described previously [31]. The restriction enzyme sequences BamHI and EcoRI were added at the DNA’s 3′ and 5′ ends, respectively. Along with this, the Kozak sequence was added to ensure efficient translational initiation in prokaryotic expression systems, which surround the starting codon. The restriction cloning module from Snapgene V.6.0.2 software was used to incorporate the multiepitope construct into the pET28a(+) plasmids using the indicated restriction enzyme sites.

### 2.8. Assessment of the Physiochemical Properties of the Designed Multiepitope H5N1 Clade 2.3.4.4b DNA Vaccine

The physicochemical properties of the designed protein were assessed using the Protparam server (https://web.expasy.org/protparam/ (accessed on 1 August 2025)). The potent antigenicity of selected proteins was predicted by using the VaxiJen v2.0 server (http://www.ddg-pharmfac.net/vaxijen/VaxiJen/VaxiJen.html (accessed on 1 August 2025)) [32] with a default threshold of 0.4. The allergenicity and toxicity of proteins were assessed by using the AllerTOP v.2.1 server (http://ddg-pharmfac.net/AllerTOP/ (accessed on 1 August 2025)) [33] and the ToxinPred2 server (https://webs.iiitd.edu.in/raghava/toxinpred2/index.html (accessed on 1 August 2025)) [34], respectively. The same server was later used to assess the physicochemical, antigenicity, allergenicity, and toxicity properties of the selection of epitopes, as well as for the designed vaccine construct. The solubility nature of the proteins, epitopes, and final vaccine construct was analyzed using the Innovagen solubility check server (http://www.innovagen.com/proteomics-tools (accessed on 1 August 2025)).

### 2.9. Prediction of the Secondary and Tertiary Structures of the Designed Multiepitope Vaccine

The secondary structure, topology, folds, and domain organization of the construct were predicted using the PDBsum server tool (https://www.ebi.ac.uk/thornton-srv/databases/pdbsum/ (accessed on 1 August 2025)) [35]. The tertiary structure was predicted using the vaccine sequence and modeled using Biovia Discovery Studio. Additionally, the ProSA server (https://prosa.services.came.sbg.ac.at/prosa.php (accessed on 1 August 2025)) was used to determine the total number of residues in the multiepitope vaccine construct. The stability was analyzed and compared through the Ramachandran plot from both Biovia Discovery Studio and the PDBSum server tool.

### 2.10. Molecular Docking of the Designed Multiepitope Vaccine Construct with the Chicken Toll-like Receptors (TLRs)

We used TLR3 and TLR7 for the molecular docking analysis with the designed vaccine construct. Hence, the full-length protein sequence of chicken TLR3 (UniProt ID: 015455) and the chicken TLR7 (UniProt ID: Q9NYK1) were retrieved from Uniprot, and their respective structure was modeled using both AlphaFold collab (https://colab.research.google.com/github/sokrypton/ColabFold/blob/main/AlphaFold2.ipynb (accessed on 1 August 2025)) and Biovia Discovery Studio for better confirmation of the structure. The active binding sites were anticipated before the docking stage because it is crucial for greater binding affinity, and the docking study between the vaccine design and TLR3/TLR7 was performed through the Biovia Discovery Studio using ZDocker. Subsequently, a detailed analysis of the binding interfaces was performed using the PDBsum server tool to characterize the molecular interactions. An analysis of protein–protein interactions was carried out using Zdocker in Biovia Discovery Studio (v22.1.021297). For the best docking analysis, several modifications should be made, including removing the water molecules, adding hydrogen, and minimizing the energy (CharmM). Out of the 10 poses generated per docking reaction, we selected the best pose, which had the highest binding energy between the target protein and its receptor, as per the default settings of the molecular docking analysis, as described in other studies [36].

### 2.11. In Silico Immune Simulation of the Designed Multiepitope H5N1 Clade 2.3.4.4b DNA Vaccine

To predict the immune response activation in response to the designed multiepitope vaccine, the in silico immune simulation was performed using the C-ImmSim server (https://kraken.iac.rm.cnr.it/C-IMMSIM/index.php (accessed on 1 August 2025)). The C-ImmSim server simulates the influence of vaccine constructs on B and T lymphocytes by modeling the immune system of the chicken’s three major immune organs (bone marrow, thymus, and spleen). The parameters were set as a default with 50 and 1000 simulation steps. We are proposing the administration of the designed multiepitope vaccine construct three times, using 4-week intervals. During simulation, each step indicates eight hours of real-time with periods of 1, 84, and 168 h. Subsequently, this server also predicts the host cellular immune response and cytokine expression levels induced by the multiepitope vaccine candidates in silico.

## 3. Results

### 3.1. Multiple Sequence Alignment and Phylogenetic Analysis of Circulating H5N1 Clade 2.3.4.4b Isolates

The HA, M2, NA, and NP proteins predicted from 279 circulating H5N1 clade 2.3.4.4b isolates were used to perform multiple sequence alignment and phylogenetic analysis. These sequences were retrieved from various avian species, including chickens, ducks, turkeys, Canadian geese, and migratory birds (Supplementary Excel Files (Tables S1–S4)). The multiple sequence alignment and phylogenetic analysis revealed a high level of conservation, with similarity levels of 99.8% for HA, 99.7% for M2, 99.6% for NA, and 99.3% for the NP proteins (Supplementary Figures S1–S4). These results indicate a high degree of genetic similarity among H5N1 clade 2.3.4.4b isolates circulating in different avian hosts.

### 3.2. Results of the Prediction of the B Cell Epitopes (Linear and Discontinuous) Within the Major Proteins of H5N1 Clade 2.3.4.4b

The B cell epitopes were analyzed from various structural proteins (HA, NA, NP, and M2) of the H5N1 clade 2.3.4.4b virus by utilizing the IEDB and BCpred server tool. We compared the results of those two servers in the prediction of the B cell epitopes using a threshold value of 0.75. Epitopes with a threshold value of 0.75 are more likely to have a higher peptide score. The IEDB server tool results showing the number of epitopes were (HA = 20), (NA = 14), (NP = 17, and M2 = 3). Results from the BCpred server tool showing the number of predicted epitopes are as follows: (HA = 442), (NA = 449), (NP = 419), and (M2 = 52). Among these peptides, the top-ranked B cell epitopes are selected based on overlapping results from the two methods, taking into consideration epitopes that show high antigenic score values, as shown in Table 1.

**Figure 2:**
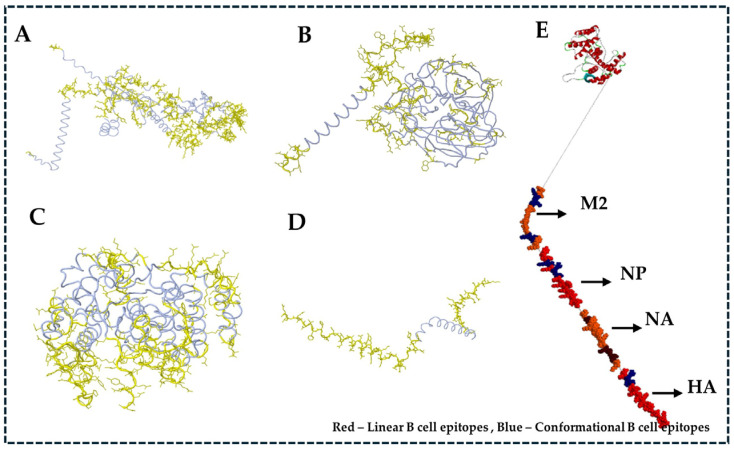
Three-dimensional structural representation of predicted B cell epitopes from H5N1 clade 2.3.4.4b structural proteins. Conformational B cell epitopes (highlighted in yellow) were identified using the Ellipro tool and are shown for (**A**) HA, (**B**) NP, (**C**) NA, and (**D**) M2 proteins. (**E**) The final multiepitope vaccine construct is visualized with linear epitopes in red and conformational epitopes in blue, mapped using BIOVIA Discovery Studio.

For the results from the Ellipro server to predict the discontinuous epitopes from the 3D structure of respective proteins, we considered a minimum score of 0.5 and a minimum distance of 6 Ǻ. The list of predicted discontinuous B cell epitopes recognized at different exposed surface areas is shown in Table 2. The position of each predicted epitope on the surface of the 3D structure of all the considered proteins of H5N1 clade 2.3.4.4b was visualized using Chimera and the Biovia Discovery Studio visualization tool (Figure 2).

### 3.3. Results of the Prediction of the Cytotoxic T Lymphocyte Epitopes (MHC Class I Molecules) Within the Major Proteins of H5N1 Clade 2.3.4.4b

Table 3 shows the predicted MHC class I epitopes with a binding affinity (IC50; IC50 < 50 nM). Table 3 also shows the parameters of the top-ranked epitopes, taking into consideration the allergenicity, antigenicity, non-toxic, and solubility per each listed epitope.

### 3.4. Results of the Prediction of the Helper T Lymphocyte Epitope Prediction Within the Major Proteins of H5N1 Clade 2.3.4.4b

Table 4 shows the predicted MHC class II epitopes with a binding affinity (IC50; IC50 < 50 nM). It shows the list of epitopes corresponding the allergenicity, antigenicity, non-toxic, and solubility properties. Our results show the predicted epitopes per each protein (HA = 13, NP = 21, NA = 2, and M2 = 9), respectively (Table 4).

### 3.5. Evaluation of the Antigenicity, Allergenicity, and Toxicity of the Predicted MHC I and MHC II Epitopes Within the Major Proteins of H5N1 Clade 2.3.4.4b (HA, NA, NP, M2)

Our analysis shows that a large number of epitopes were identified; we then filtered and ranked these epitopes based on a percentile rank < 4 and their IC50 value < 50 nM in the case of the MHC class I (Table 3) and MHC class II molecules (Table 4). The top-ranked antigenic epitopes shown in Table 3 and Table 4 for each class of the MHC molecules were further evaluated for their potential allergenicity and toxicity. Finally, epitopes showing better solubility and stability were considered and ranked, as shown in Table 5.

### 3.6. Results of the Molecular Docking of the Selected MHC Class I and II Epitopes with the Chicken Alleles

The molecular docking analysis was performed by docking MHC class I and II molecules with chicken alleles (BF2 * 2101—for MHC class I and Gaga_BLB1 and Gaga_BLB2—MHC class II) using the HADdock server tool, using peptide-binding groove affinity. We used chicken alleles as receptors, and the MHC class I and MHC class II peptides listed in Table 3 and Table 4 were considered as ligands. Results show the binding affinity and confidence score as listed in Table 4. The top-ranked epitopes showing the highest binding affinity score were chosen for the design of the final vaccine construct, as listed in Table 5.

**Figure 3:**
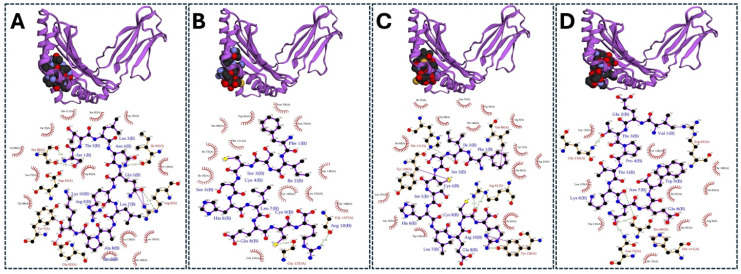
Three-dimensional structure representation and interaction analysis of high-ranked MHC class I epitopes from the H5N1 clade 2.3.4.4b structural proteins docked with the chicken MHC allele BF22101. Top panels (**A**–**D**) show the molecular docking results of selected epitopes with BF22101 using the HADDOCK server: (**A**) HA epitope: KVRLQLRDNA, (**B**) NP epitope: FQGRGVFEL, (**C**) NA epitope: FISCSHLECR, and (**D**) M2 epitope: VYRRLKYGLK. Bottom panels present the respective interacting residues as predicted by the PDBsum analysis.

**Figure 4:**
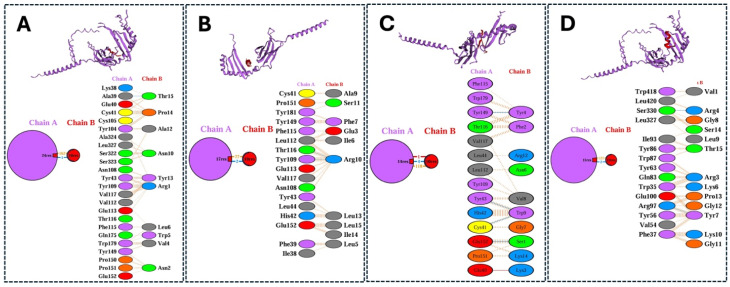
The 3D structure representation (top) of the molecular docking analysis of top-ranked MHC class II epitopes from different structural proteins (HA, NP, NA, and M2) of H5N1 clade 2.3.4.4b with chicken alleles BLB1 and BLB2 using the HADdock analysis server, (**A**) HA: RNVVWLIKKNDAYPT, (**B**) NP: EIEDLIFLARSALIL, (**C**) NA: FKYGNGVWIGRTKS, (**D**) M2: VYRRLKYGLKGGPST, and their respective interaction residues (bottom) obtained from the PDBsum database.

The interaction residues from those docking results of MHC class I with the chicken MHC allele BF2 * 2101 and MHC class II molecules with chicken alleles BLB1 and BLB2 were analyzed using the PDBsum server tool and are shown in Figure 3 and Figure 4. Both the analyzed data illustrate the interaction/binding affinities between the amino acids of the epitopes and their respective protein structures (chicken alleles).

### 3.7. The Structure and Design of the Multiepitope DNA-Based Vaccine Against H5N1 Clade 2.3.4.4b Spanning Top-Ranked Epitopes Within the Four Major Viral Proteins (HA, NA, NP, and M2)

**Figure 5:**
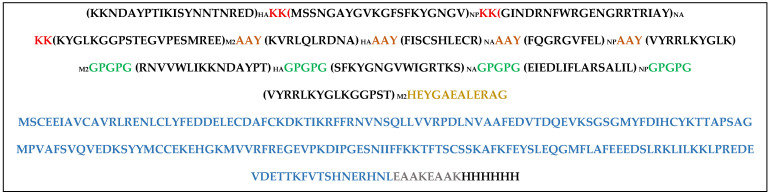
Nucleotide sequence of the designed multiepitope DNA vaccine construct incorporating top-ranked epitopes derived from major structural proteins (HA, NA, NP, and M2) of H5N1 clade 2.3.4.4b. The constructs were designed to optimize expression and immunogenicity in avian hosts.

We designed the final vaccine construct by combining the top-ranked B cell epitopes and T cell epitopes of both MHC I and MHC II classes of molecules, filtered from high antigenic, non-allergic, non-toxic and good solubility, with a better binding affinity score of the structural proteins (HA, NP, NA, and M2) of H5N1 clade 2.3.4.4b, as listed in Table 5. The top-ranked B cell and T cell epitopes were linked by using the KK, GPGPG, and AAY as linkers, respectively, whereas the C-terminal ends of the vaccine construct were linked to the full-length chicken IL-18 gene (Accession No. CAB96214) as an adjuvant after separation with the PEAK linker (Figure 5). Additionally, we incorporated the 6 × His-tag (HHHHHH) attached to the C-terminus for the purification and identification of the vaccine upon expression.

**Figure 6:**
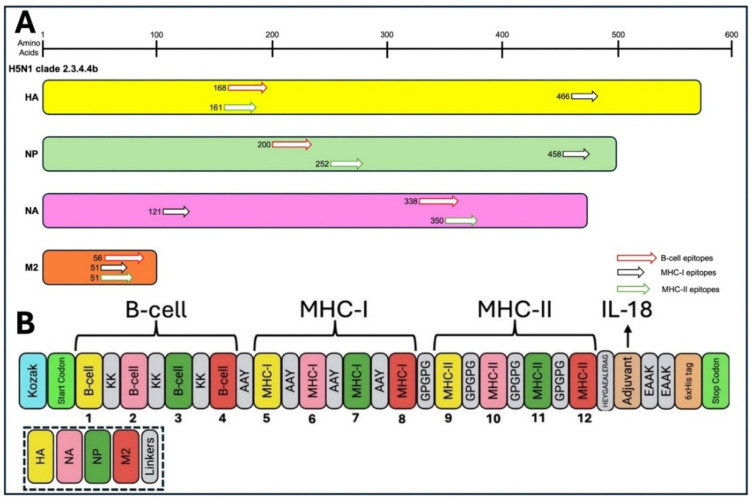
Schematic representation of the multiepitope DNA vaccine construct targeting top-ranked epitopes from the major structural proteins (HA, NA, NP, and M2) of the currently circulating H5N1 clade 2.3.4.4b in chickens. (**A**) Mapping of predicted B cell, MHC class I, and MHC class II epitopes across viral proteins. (**B**) Design of the final multiepitope vaccine construct comprising twelve selected epitopes genetically linked to the chicken IL-18 gene to enhance immunogenicity.

The final vaccine construct (Figure 6) is designed as follows: the B cell epitopes are shown in purple, linked with KK; the MHC-I T cell epitopes are shown in green, linked with AAY; and the MHC-II T cell epitopes are shown in orange, linked with GPGPG. The linkers are shown in bold letters, and the adjuvant (IL-18) is shown in red. MHC II is linked with adjuvant using HEYGAEALERAG. The IL-18 adjuvant is linked with a 6 × His tag using EAAAK.

### 3.8. D Structural Comparison and Comparative Epitope Mapping with Monoclonal Antibodies Targeting HA

**Figure 7:**
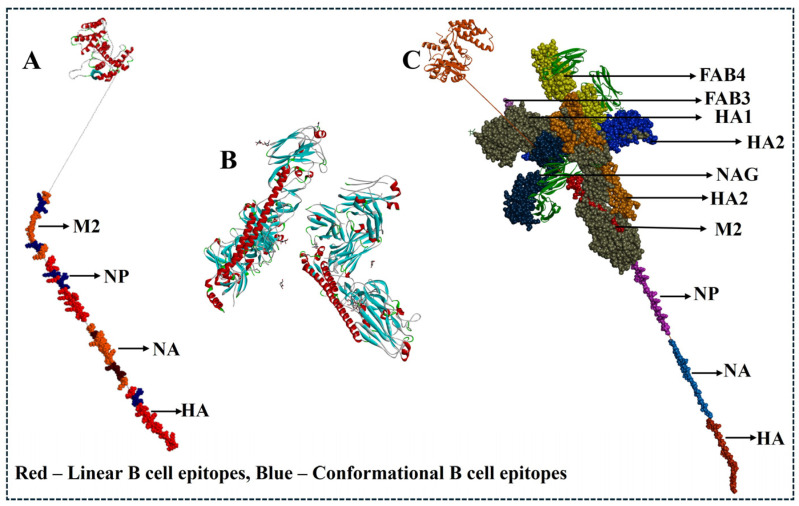
Schematic and structural representation of the multiepitope DNA vaccine construct and its immunological relevance against H5N1 clade 2.3.4.4b in chickens. (**A**) Design of the multiepitope DNA vaccine incorporating top-ranked epitopes from major viral proteins (HA, NA, NP, and M2). (**B**) Crystal structure of the Fab CR6261 monoclonal antibody in complex with H5N1 influenza virus hemagglutinin (PDB ID: 3GBM), illustrating a known neutralizing epitope. (**C**) Structural comparison and mapping of both linear and conformational B cell epitopes from the final vaccine construct onto the HA protein, highlighting regions targeted by neutralizing antibodies.

**Figure 8:**
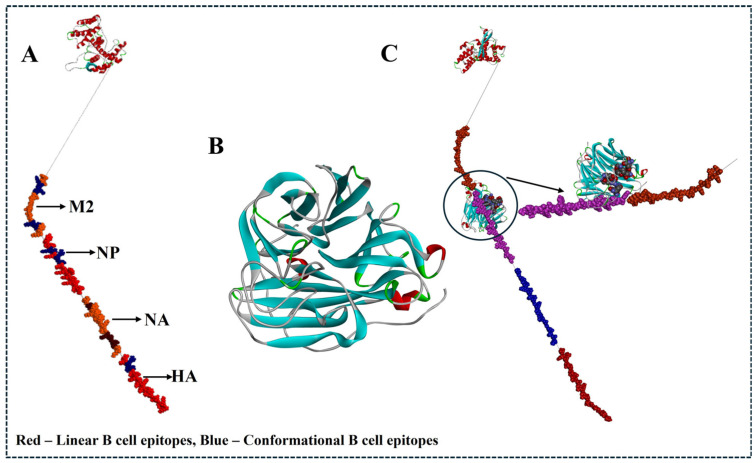
Schematic and structural representation of the multiepitope DNA vaccine design and its epitope mapping on neuraminidase (NA) of H5N1 clade 2.3.4.4b. (**A**) Design of the multiepitope DNA vaccine incorporating top-ranked epitopes from major structural proteins (HA, NA, NP, and M2) of circulating H5N1 strains in chickens. (**B**) Crystal structure of influenza A virus neuraminidase, specifically the N1 subtype from H5N1 (PDB ID: 2HTY), representing group 1 NA enzymes. (**C**) Structural comparison and mapping of linear and conformational B cell epitopes from the final vaccine construct onto the NA protein, illustrating their overlap with known neutralizing antibody-binding regions.

The homology model of the final multiepitope vaccine construct was generated using Biovia Discovery Studio, and its stability was analyzed using the Ramachandran Plot. The structural comparison/superimposition was performed between the final vaccine construct and HA monoclonal antibody complexes (CR6261) (PDB ID: 3GBM) (Figure 6) and for NA monoclonal antibody-bound viral epitopes (PDB ID: 2HTY)—Figure 7. We initially loaded and superimposed both PDB structures using Chimera and mapped the predicted B cell epitopes (linear and conformational) and experimentally validated epitope residues recognized by the monoclonal antibody using Biovia Discovery Studio. The analysis of conformational epitope residues leads to the prediction of native-like folding and an active immune response (Figure 7 and Figure 8).

### 3.9. Results of the Physiochemical Properties of the Designed Multiepitope DNA-Based Vaccine Against H5N1 Clade 2.3.4.4b

The predicted vaccine weighed 49,942.78 Dalton and possessed a theoretical isoelectric point of 9.04, indicating the alkaline nature of the constructed vaccine. The total number of negative and positively charged residues was 57 and 70, and the extinction coefficient measured at 280 nm in water was shown to be 51,395, assuming all pairs of Cys residues form cystines. The instability index (II) was about 36.74, showing that the structure of the vaccine protein was stable. The aliphatic index was about 65.19, indicating the hydrophilic nature with a value of −0.596.

### 3.10. Results of the Secondary and Tertiary Structures of the Designed Vaccine Construct

**Figure 9:**
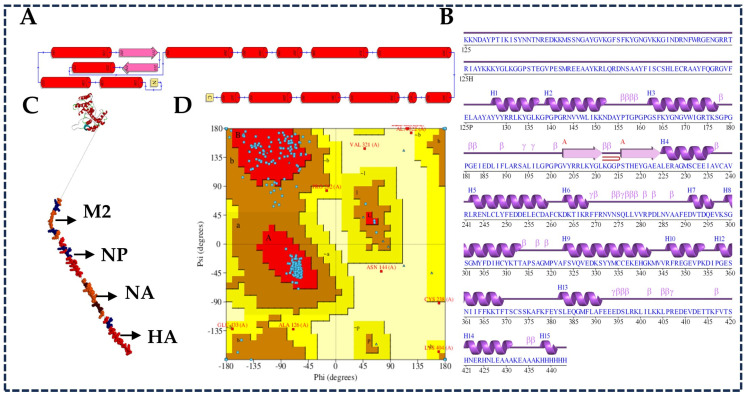
Structural analysis of the final multiepitope vaccine construct against H5N1 clade 2.3.4.4b: (**A**) shows the multiepitope vaccine construct’s topology diagram to visualize the secondary structure elements’ arrangements. The PDB files of the vaccine construct were provided as input for the PDBsum server; the results showed the cylinders, arrows, and lines, which represent the alpha-helix and beta strands, and the lines explain the connection via loops and chains. (**B**) The secondary structure prediction exactly matches the topology diagram, allowing the identification of the flexible regions, surface exposure, and potential antigenic sites; (**C**) shows the 3D structure of the vaccine construct modeled through Biovia Discovery Studio using the template sequence alignment method, and its corresponding Ramachandran plot (**D**) confirms the stability, with a greater number of blue dots on the respective region, confirms the quality of protein conformations, and ensures its accuracy.

The secondary and tertiary structures of the multiepitope-based vaccine construct were analyzed and modeled through the PDBsum server tool and Biovia Discovery Studio (Figure 9).

### 3.11. Visualization of B Cell and T Cell Epitopes from the Final Vaccine Construct with Its Native Proteins

**Figure 10:**
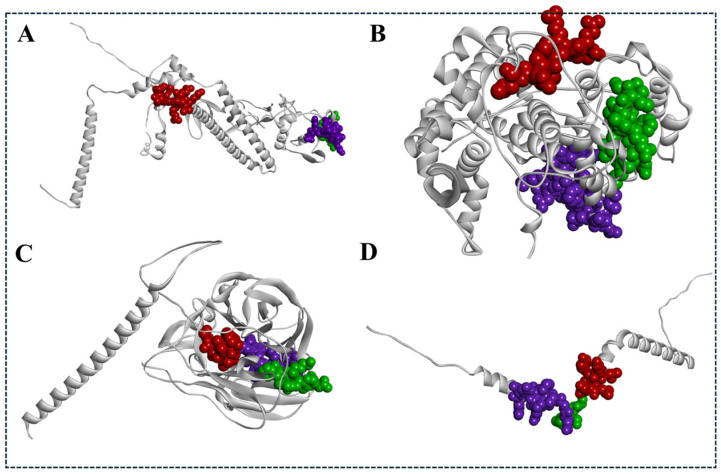
Visualization of top-ranked predicted epitopes mapped onto their respective native H5N1 clade 2.3.4.4b proteins using the Dassault Systèmes BIOVIA Discovery Studio. B cell epitopes are shown in purple, MHC class I epitopes in red, and MHC class II epitopes in green. Panels display epitope localization on (**A**) Hemagglutinin (HA), (**B**) nucleoprotein (NP), (**C**) neuraminidase (NA), and (**D**) matrix protein 2 (M2).

Figure 10A–D visualizes the predicted B cell and MHC class I and II of T cell top-ranked epitopes of all structural proteins, such as HA, NP, NA, and M2, through the Dassault system Biovia Discovery Studio, which involves the complete analysis and optimization of the structural features of the vaccine design. Initially, it starts with importing the desired protein structures using their respective PDB files. The epitope sequences, identified through different computational tools, were aligned, and the sequence alignment tool from the Discovery Studio allows us to map this sequence with its desired corresponding regions.

### 3.12. Results of the Molecular Docking of the Designed Vaccine Construct with the Chickens’ Toll-like Receptors (TLR3 and TLR7)

**Figure 11:**
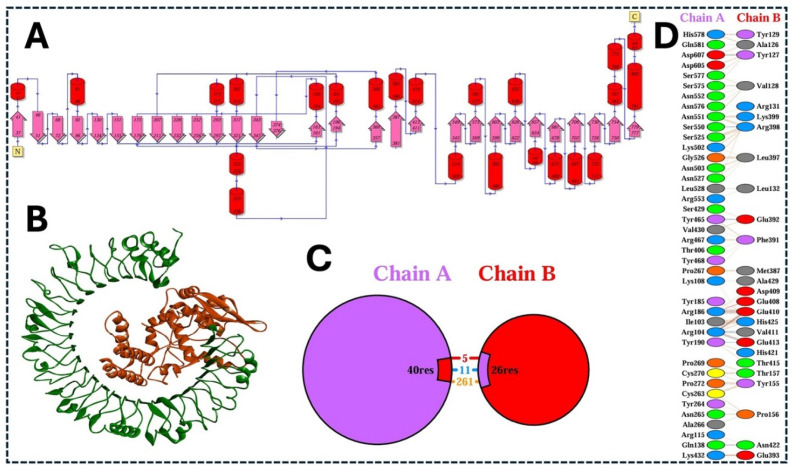
Molecular docking analysis of the multiepitope vaccine construct targeting H5N1 clade 2.3.4.4b with the chicken Toll-like receptor 3 (TLR3) using BIOVIA Discovery Studio. (**A**) Topology diagram of the chicken TLR3 protein structure generated via the PDBsum server. (**B**) Docked complex showing the binding interaction between the vaccine construct and TLR3. (**C**) Visualization of the interacting residues at the interface of the vaccine construct and TLR3. (**D**) List of binding amino acid residues involved in the interaction, as identified through PDBsum analysis.

To examine the potential immunogenic performance of a multiepitope-based vaccine construct combining four proteins (HA, NA, NP, and M2) of H5N1 clade 2.3.4.4b, molecular docking studies were performed between the vaccine construct and Toll-like receptors (TLR3 and TLR7). As mentioned earlier, TLR3 and TLR7 were chosen among the ten toll-like receptors because these intracellular receptors can trigger an innate immune response through several pathways. Initially, the sequences of TLR3 (Uniprot ID: QoPQ88) and TLR7 (Uniprot ID: C4PCM1) were retrieved from the database and modeled using Biovia Discovery Studio. This was followed by preparing the proteins for the docking study by removing water molecules, adding hydrogen atoms, and performing energy minimization. Docking analysis was performed using the Zdocker, and the results obtained indicate a strong binding affinity between the vaccine construct and Toll-like receptors (TLR3 and TLR7). The best-ranked complexes, along with their respective ZDock scores, provide confirmation of the firm and stable interaction between them. The interaction residues, multiple hydrogen bonds, and hydrophobic bonds were analyzed through the PDBsum server tool. Figure 11 and Figure S5A show the topology visualization of TLR3 and TLR7, Figure 11 and Figure S5B show the docking interaction analysis between the vaccine construct and Toll-like receptors, and finally, Figure 11 and Figure S5C illustrate the interaction of amino acid residues and the formation of multiple hydrogen bonds, hydrophobic bonds, etc., through the PDBsum server tool.

### 3.13. In Silico Cloning of the H5N1 Clade 2.3.4.4b Multiepitope-Based Vaccine Spanning Key Epitopes Within the Major Proteins (HA, NA, NP, and M2) 

The vaccine construct was cloned using the Vector builder from the decoded amino acid sequence of each epitope’s respective DNA sequences to mimic the vaccine’s expression in the *E. coli* K12 expression vector. The GC content and codon adaptation index values generated by the Vector builder server represent the level of expression in the *E. coli* system. Finally, snapgene software was used to clone the constructed vaccines into the pET-28a(+) expression vector between the restriction enzyme cutting locations of BamHI and EcoRI, and the results obtained are shown in Figure S6.

### 3.14. In Silico Immune Simulation of the Designed H5N1 Clade 2.3.4.4b Multiepitope-Based Vaccine Spanning Key Epitopes Within the HA, NA, NP, and M2 Proteins

The predicted immune response of the constructed vaccine was analyzed through the interaction between the H5N1 clade 2.3.4.4b antigens and the B cell, T cell, and cytokines, as shown in Figure S7.

## 4. Discussion

The HPAIV-H5N1 clade 2.3.4.4b belonging to the highly pathogenic avian influenza virus has become a serious hazard to poultry populations on several continents as of 2020 [32,33]. Clade 2.3.4.4b, which has resurfaced in recent years, seems to be present in a range of bird species, such as ducks, Canadian geese, turkeys, etc. Along with this, several cases of cross-species transmission, including rare infections, have been reported in humans as well as in mammals, including dairy cows, mink, cats, foxes, and sea lions [37,38]. As it is causing major concern for both animal health and food security, there is an urgent need to develop effective vaccines that can protect chickens and other birds against this highly pathogenic, emerging virus. The application of AI tools in vaccine design and development has increased significantly over the past five years [39,40]. AI tools, including epitope prediction, molecular docking, and simulation, paved the way for a remarkable short-term development of vaccine pipelines for many viral diseases affecting domestic animals and birds [36,41].

Several traditional methods were employed for epitope mapping across various viral genomes. The application of monoclonal antibodies (mAbs) was used in the past and may still be in use as a conventional method for epitope mapping for H5N1 for a decade. The mAbs approach requires the use of animals and is time-consuming and labor-intensive [42]. This contrasts with the application of AI in the prediction and simulation of protein/protein interactions, which is very efficient, fast, and has a high level of accuracy and precision. Specifically, we used AI-based algorithm tools such as Alpha fold 2 and Biovia Discovery Studio for modeling 3D structures of our final vaccine constructs targeting four key antigenic structural proteins (HA, NA, NP, and M2) of the chicken influenza virus. Along with this, in silico cloning and codon optimization techniques were used to improve the expression and effectiveness of the candidate vaccines in the prokaryotic expression system.

In the current study, we used several AI tools to design a multiepitope DNA-based vaccine against the currently circulating clade H5N1 2.3.4.4b in chickens. Furthermore, the characteristic features, such as the antigenicity, allergenicity, and structural validation of the designed vaccine, were analyzed in parallel with molecular docking and in silico simulation, which provided a pathway for eliciting strong cellular and humoral immune responses. [43]. Our approach for the design of the multiepitope DNA-based vaccine against the currently circulating H5N1 clade 2.3.4.4b includes several consecutive steps, including (1) retrieval of the sequences from the GenBank (2) multiple sequence alignment, (3) generation of the consensus sequences per each protein, (4) prediction of the B cell and T cell including MHC class (I and II), (5) selection of the top-ranked epitopes, (6) construction of the multiepitope using the appropriate linkers, (7) incorporation of the IL18 to the vaccine construct, (8) in silico cloning of the designed vaccine, (9), prediction of the physicochemical properties of the designed vaccine, (10) prediction of the secondary and tertiary structures of the designed vaccine, (11) molecular docking of the designed vaccine with the chickens TL3/TLR7, and (12) immunosimulation of the final vaccine construct to assess its potential potency in the activation of the humoral and cell-mediated immunity of chickens. Our prediction shows many potential epitopes per protein. We established some filtration criteria to select the top-ranked epitope per category of immunogens. First, we used the percentile score (<4) for MHC class I molecules and (<10) for MHC class II molecules, with an IC50 value of (<50 nM). Second, the short-listed epitopes per protein were examined for their allergenicity, antigenicity, non-toxicity, and solubility profiles as previously described [44,45]. Third, we used VaxiJen 2.0 and AllerTop to assess the antigenic properties and allergic nature of each candidate epitope. The acceptable antigenic score range was established to be 0.4–0.5. Fourth, we tested all the short-listed epitopes for potential toxicity using the ToxinPred server tools, as previously described [46,47]. Fifth, the molecular docking analysis was performed between the filtered epitopes and chicken alleles of MHC class I and MHC class II molecules through the HADdock docking tool.

The top-ranked peptide was selected based on its binding score and high antigenic score for all the structural genomes we considered for the study, for MHC class I of molecules—KVRLQLRDNA (1.5926 and −188.17 docking score—HA), FQGRGVFEL (1.2783 and −214.75 docking score—NP), FISCSHLECR (1.0798 and −214.75 docking score—NA), VYRRLKYGLK (1.2596 and −178.50 docking score—M2)—and for MHC class II of molecules—RNVVWLIKKNDAYPT (1.2023 and docking score of −263.89—HA), EIEDLIFLARSALIL (0.9266 and docking score of −214.79—NP), SFKYGNGVWIGRTKS (1.2583 and docking score of −255.61—NA) and VYRRLKYGLKGGPST (1.2088 and docking score of −249.52—M2) [22]. Finally, these epitopes were used in the vaccine construct and were designed using linkers and adjuvants. Chicken IL-18 was used as an adjuvant in our vaccine construct. It is a crucial cytokine that plays an important role in host innate and adaptive immunity. Chicken IL-18 has been used as an adjuvant in several previous vaccine constructs, including those against IBDV [30,48], NDV [29], and H5 influenza virus [49]. The interaction residues between them were identified through PDBsum and are displayed in the figure, resulting in multiple hydrogen bonds and hydrophobic bonds, especially to capture their better binding interactions. And for the selection of B cell epitopes for the multiepitope vaccine construct, we carried out the initial choice of B cell epitopes based on linear epitope prediction tools (IEDB, Bepipred), and parallelly, we employed Ellipro to identify conformational B cell epitopes from the 3D structure of native viral proteins such as HA, NA, NP, and M2. Upon mapping these conformational epitopes, we found that several residues overlapped with or were near the linear epitopes included in the multiepitope vaccine (MEV) construct. This overlap suggests that the selected regions include properties of both linear and conformational B cell epitopes, which were then carried over into the final MEV design. To preserve the native-like conformation of conformational epitopes in the MEV, we used flexible linkers (GPGPG and EAAAK), performed 3D structure prediction with AlphaFold2 and Biovia Discovery Studio, and verified the model using energy minimization, Ramachandran analysis, and surface epitope mapping. These processes guaranteed that the epitope areas folded properly, were solvent-accessible, and had structural integrity.

Previous studies have shown experimentally that the multiepitope vaccine strategy represents a promising approach against several avian influenza viruses. For instance, a recent study showed that the region at proteins NS_198-106_, PB2_552-560_, NP_182-190_, and NP_455-463_, which are conserved across both H7N9 and H9N2 of AIV, have been proven as CD8 T cell epitopes that induced an immune response in the chicken B2 haplotype in vivo [50]. Another study reported that an adenovirus-based multiepitope vaccine from relatively conserved immunogenic domains of H5N1, including (M2 ectodomain), HA fusion domain, T cell epitopes of nucleoprotein, and HA alpha-helix domain, caused significant inhibition of viral load in the lungs of mice challenged with H5, H7, and H9 avian influenza viruses [51].

One of the challenges in this study is that data on the epitopes interacting with chicken MHC-I and MHC-II are not yet available on the IEDB server. To overcome this problem, we used alternative strategies to try to identify epitopes activating chicken CTL and HTL. We applied the surrogate model approach using the well-known human alleles because there are not many computational tools available, specifically for MHC class I molecules of most avian species, particularly chickens. Both the human and chicken alleles are very similar in their structural and functional properties, including the peptide-binding grooves, which enable the peptide–MHC class molecules’ binding interactions. We selected the human alleles that match the chicken alleles’ structural and functional properties through the IEDB.org server and performed the prediction. The default parameter setting was kept the same as the polymerase length of 12 mer. Despite the species-specific diversity of the chicken MHC class I molecules from the BF2 locus, the experimental data found in the IEDB MHC class I molecules server (https://www.iedb.org/ (accessed on 1 August 2025)) have confirmed that specific BF2 alleles, like BF2 * 2101 from previous studies, have similarities to human alleles (HLA—A02:01), especially in the motif-binding and anchor residue preferences.

Regarding the prediction of the helper T-lymphocyte epitopes (MHC class II molecules), we used the MixMHC2pred tools (http://mixmhc2pred.gfellerlab.org/ (accessed on 1 August 2025)) to predict the corresponding chicken alleles (Gaga_BLB1_002_01, Gaga_BLB1_012_01, Gaga_BLB2_002_01, Gaga_BLB2_012_01, and Gaga_BLB2_012_02) for the selected list of epitopes. This approach successfully provided the best score data and matched the chicken alleles with the corresponding epitopes.

Furthermore, to validate our vaccine construct, a comparison study was performed between the final vaccine construct and the monoclonal antibody targeting HA (CR6261 -PDB ID: 3GBM) [52,53,54,55] and for the NA monoclonal antibody-bound viral epitopes (PDB ID: 2HTY) [56]. As we all know, among all other structural proteins, HA is the major surface glycoprotein of the influenza virus, which is directly responsible for virus attachment and entry into the host cell. In the immune system, B cells specifically target HA to inhibit virus entry; hence, the evaluation of our final vaccine construct with experimentally validated monoclonal antibody targeting HA is necessary. Initially, we loaded and superimposed both of the PDB structures using Chimera and mapped the predicted B cell epitopes (linear and conformational) with Biovia Discovery Studio. The results from Biovia Discovery Studio showed that the multiepitope vaccine construct was not identical in sequence, but we observed several regions that were spatially adjacent to the antibody-binding site on HA and NA. This shows that the multiepitope vaccine construct may maintain structural properties like linear and conformational B cell epitopes, which supports its ability to induce a neutralizing antibody response. And the surface rendering, solvent accessibility analysis, and RMSD-based comparison results from Chimera showed epitope structural integrity and exposure (sequence alignment score of 99.2, with a local RMSD of ~0.402 Å for HA (three trimmed regions) and sequence alignment score of 79.9, with a local RMSD of ~0.855 Å between four trimmed regions for NA), indicating substantial spatial similarity [57,58,59,60].

A recent in silico study demonstrated that a multiepitope DNA vaccine candidate against infectious bronchitis virus exhibited strong immune simulation results and showed stable binding affinity to both TLR3 and TLR7 receptors [61]. Our docking simulation results showed the firm binding affinities between the designed vaccine epitopes and the conjugated TLRs, facilitating effective immune recognition and the initiation of a robust immune response [62]. The Z-score identifies several high-affinity binding poses in the molecular docking results of the designed multiepitope-based vaccine construct of H5N1 clade 2.3.4.4b. The high-accuracy protein–protein docking resulted in the formation of multiple hydrogen bonds and hydrophobic interactions with the Zdock score (18), Zrank score (131.75), and E_Rdock score (−7.95511) for TLR3 and the Zdock score (19.04), Z-rank score (−142.71), and E_Rdock score (−45.31) for TLR7. The PDBsum results showed that the interaction residues were analyzed and the major interaction hydrogen bonds (Arg65—Thr419), (His 109—Cys261), (Ser133—Asp263), and (Lys331—Tyr310) for TLR3 with the vaccine construct and the major interaction hydrogen bonds (SER550—Arg398), (Arg 186—Glu 408), (Arg 104—Val 411), and (Tyr 190—Glu 413) for TLR7 with the vaccine construct.

In silico immune simulations using C-ImmSim provided critical insights into the potential immune responses elicited by the four designed structural proteins (HA, NP, NA, and M2) of the H5N1 clade 2.3.4.4b vaccine constructs. Our approach ensured that vaccination candidates had reliable protein synthesis and effective translation, utilizing optimized codons and computational tools such as Vector builders and SnapGene [63]. The simulation results revealed the robust activation of T cell populations, including cytotoxic T cells and helper T cells, crucial for cellular and humoral immunity [64]. This comprehensive analysis demonstrated that the multiepitope H5N1 vaccine constructs in this study would induce strong humoral and cell-mediated immunity, which might play essential roles in protecting chickens and other bird species against the currently circulating HPAI-H5N1 clade 2.3.4.4b.

Based on the data provided above, a high level of humoral immune response (immunoglobulin antibodies) and other immune cells are expected after the administration of the candidate vaccines [23,65]. It also predicted the progression of the magnitude of the immune response with the progression of time after administering these candidate vaccines (primary immune response). We think our designed multiepitope-based vaccine spanning the four major structural proteins (HA, NP, NA, and M2) of H5N1 clade 2.3.4.4b will be effective in the protection of birds against the currently circulating clade of the H5N1 in chickens in the USA and other parts of the world. However, further studies are required to validate these vaccines in chickens. And moreover, we agree with the importance of experimental validation; hence, our next step would be the synthesis of the optimized DNA construct and validating its properties in chicken models through in vitro and in vivo studies. When we compare the advantages of our AI-derived epitope design with the large-scale experimental techniques, though the experimental methods offer incredible evidence of epitope presentation, it ends up utilizing large-scale resources and limits its advantage due to the lack of information available for the MHC classes of molecules for avian species. In contrast, our approach is rapid, cost-effective, and allows for high-throughput screening of antigenic regions across multiple proteins and strains. Along with this, the integration of safety profiling, host-specific MHC prediction, and codon optimization enhances its advantage. Hence, AI-derived computational tools would serve as the pathway to accelerate epitope-based vaccine discovery, especially in contexts where experimental data are limited or emerging pathogens are involved.

## 5. Conclusions

We successfully designed a multiepitope Pan-H5N1 clade 2.3.4.4b DNA-based vaccine spanning the top-ranked immunogenic, non-allergenic, and non-toxic epitopes. Twelve epitopes within the major proteins (HA, NA, NP, and M2), including B cell, MHC class I, and MHC class II. The T cell epitopes showed high binding affinities with the chicken alleles. We successfully made silicon cloning of these epitopes and linked them to the chicken IL-18. The designed vaccine construct showed high binding affinities to the chicken Toll-like receptors 3 and 7. The designed vaccine construct showed high immunogenic potential in terms of the production of humoral and cell-mediated immunity in chickens using an immune simulation approach. We believe the designed vaccine in the current study will protect not only chickens but also other birds, such as turkeys, quails, pheasants, and wild birds, against the currently circulating HPAIV-H5N1 clade 2.3.4.4b. The limitations of the predicted multiepitope-based vaccine are that, even though it exhibits promising immunogenic and structural characteristic features through computational analysis, it is important to understand that it is completely based on utilizing various computational prediction tools. But as we all know, the structural conformation and their recognition by immune receptors may vary in biological systems due to the absence of cellular context, post-translational modifications, and protein processing mechanisms. Our future work will focus on validating those multiepitope-based vaccine constructs through in vitro expression, antigenicity assays, and animal model studies.

## Figures and Tables

**Table 1:** List of the top-ranked antigenic B cell epitopes across the H5N1 clade 2.3.4.4b proteins (HA, NA, NP, and M2) and their information.

Starting Position	Epitope Prediction		Score	Antigen/Non-Antigen Property
	IEDB	BCpred		
**HA protein**				
168	KKNDAYPTIKISYNNTNR	KKNDAYPTIKISYNNTNRED	0.897	1.1073
221	STLNQRLAPKIATRSQVNGQRGINSSMPFHNI	LNQRLAPKIATRSQVNGQRG	0.825	1.0247
270	RNSPLREKRRKR	ATGLRNSPLREKRRKRGLFG	0.828	0.9293
**NP protein**				
5	GTKRSYEQMETGGERQNATE	GTKRSYEQMETGGERQNATE	0.985	0.5451
200	GINDRNFWRGENGRRTRIAY	RNFWRGENGRRTRI	0.757	0.9417
345	SFIRGTRVVPRGQLSTERAT	RGTRVVPRGQLS	0.743	0.4891
**NA protein**				
33	WVSHSIQTGNQYQPEPCNQS	QTGNQYQPEPCNQS	0.892	0.6502
209	NGIITDTIKSWRNNILRTQE	TDTIKSWRNNILRT	0.836	0.5221
338	MSSNGAYGVKGFSFKYGNGV	GNGV	0.77	0.9688
**M2 protein**				
6	EVETPTKNEWECNCSDSSDP	EVETPTKNEWE	0.976	0.7082
56	KYGLKGGPSTEGVPESMREE	KYGLKGGPSTEGVPESMREEYRQEQQSAVDVDDGHFV	0.918	0.8569
72	MREEYRQEQQSAVDVDDGHF	KYGLKGGPSTEGVPESMREEYRQEQQSAVDVDDGHFV	0.87	0.8804

**Table 2:** List of the structure-based prediction of the discontinuous B cell epitopes across the H5N1 clade 2.3.4.4b proteins (HA, NA, NP, and M2) and their information.

Predicted Discontinuous Epitope(s)				
No.	Protein	Peptide	No. of Residues	Score
1	HA	A:I505, A:C506, A:I507	3	0.993
2		A:E2, A:N3, A:I4, A:V5, A:L6, A:L7, A:L8, A:A9, A:I10, A:V11, A:S12, A:L13, A:V14, A:K15, A:S16, A:D17, A:D405, A:K406, A:V407, A:R408, A:L409, A:Q410, A:L411, A:R412, A:D413, A:N414, A:A415, A:E424, A:F425, A:Y426, A:H427, A:K428, A:C429, A:D430, A:N431, A:E432, A:C433, A:M434, A:E435, A:S436, A:V437, A:R438, A:N439, A:G440, A:T441, A:Y442, A:D443, A:Y444, A:P445, A:Q446, A:Y447, A:S448, A:E449, A:E450, A:A451, A:R452, A:L453, A:K454, A:R455, A:E456, A:E457, A:I458, A:S459, A:G460, A:V461, A:K462, A:L463, A:E464, A:S465, A:V466, A:G467, A:T468, A:Y469, A:Q470, A:I471, A:L472, A:S473, A:I474, A:S476, A:T477, A:A478, A:A479, A:S480, A:S481, A:L482, A:A483, A:L484, A:A485, A:I486, A:M487, A:M488, A:A489, A:G490, A:L491, A:S492, A:L493, A:W494, A:M495, A:C496, A:S497, A:N498, A:G499, A:S500, A:L501, A:Q502, A:C503	106	0.831
3		A:K177, A:I178, A:S179	3	0.721
		A:L105, A:C106, A:Y107, A:P108, A:G109, A:F127, A:E128, A:K129, A:I130, A:L131, A:I132, A:I133, A:P134, A:K135, A:S136, A:S137, A:W138, A:P139, A:N140, A:H141, A:E142, A:T143, A:S144, A:L145, A:G146, A:V147, A:S148, A:A149, A:A150, A:C151, A:P152, A:G155, A:A156, A:P157, A:S158, A:F159, A:F160, A:V163, A:V164, A:W165, A:L166, A:I167, A:K168, A:K169, A:N170, A:D171, A:A172, A:Y173, A:P174, A:T175, A:I176, A:Y180, A:N181, A:N182, A:T183, A:N184, A:E186, A:D187, A:L188, A:L189, A:W192, A:G193, A:I194, A:H195, A:H196, A:S197, A:N198, A:N199, A:A200, A:E201, A:E202, A:Q203, A:T204, A:N205, A:L206, A:Y207, A:K208, A:N209, A:P210, A:T211, A:T212, A:Y213, A:I214, A:S215, A:V216, A:G217, A:T218, A:S219, A:T220, A:L221, A:N222, A:Q223, A:R224, A:L225, A:A226, A:P227, A:K228, A:I229, A:A230, A:T231, A:R232	101	0.676
4		A:N357, A:L358, A:I362, A:N364, A:L365, A:K368		
5		A:N357, A:L358, A:I362, A:N364, A:L365, A:K368	6	0.582
6		A:N313, A:E314, A:Q315	3	0.579
7		A:G286, A:L287, A:F288, A:G289, A:A290, A:I291, A:A292, A:G293, A:F294, A:I295, A:E296, A:G297, A:G298, A:W299, A:M302	15	0.533
8		A:D70, A:G79, A:N80, A:P81, A:M82, A:D84, A:I87, A:N100, A:P101, A:A102, A:N103, A:Y153, A:Q154, A:R161, A:S233, A:Q234, A:V235, A:N236, A:G237	19	0.531
1	NA	A:R99, A:D101, A:G102, A:K103, A:W104	5	0.892
2		A:R8, A:S9, A:E11, A:Q12, A:E14, A:T15, A:G16, A:G17, A:E18	9	0.865
3		A:G200, A:I201, A:N202, A:D203, A:N205, A:F206, A:W207, A:R208, A:G209, A:E210, A:N211, A:G212, A:R213, A:R214, A:T215	15	0.856
4		A:D420, A:M421, A:S422, A:N423	4	0.85
5		A:M1, A:A2, A:S3, A:Q4, A:G5, A:T6, A:K7	7	0.739
6		A:G402, A:V403, A:F404, A:E405, A:L406, A:T407, A:D408, A:E409, A:K410, A:A411, A:T412, A:N413, A:P414, A:I415, A:V416, A:P417, A:S418, A:F419	18	0.729
7		A:R216, A:I217, A:E220, A:T232, A:A233, A:A234, A:A237, A:D240, A:Q241, A:R243, A:E244, A:S245, A:N247, A:P248, A:G249, A:N250, A:A251, A:E252, A:E254, A:I265, A:R348, A:G349, A:T350, A:V352, A:V353, A:P354, A:G356, A:Q357, A:L358, A:S359, A:T360, A:E361, A:A363, A:T364, A:I365, A:M366, A:A367, A:A368, A:F369, A:T370, A:G371, A:N372, A:T373, A:E374, A:G375, A:R376, A:T377, A:S378, A:D379, A:M380, A:R381, A:T382, A:E383, A:I384, A:I385, A:R386, A:M387, A:M388, A:E389, A:N390, A:A391, A:R392, A:P393, A:E394, A:D395	65	0.724
8		A:Q42, A:T45, A:E46, A:L47, A:K48, A:L49, A:S50, A:D51, A:Y52, A:E53, A:R55, A:F71, A:D72, A:N76, A:K77, A:Y78, A:L79, A:E80, A:E81, A:H82, A:P83, A:S84, A:A85, A:G86, A:K87, A:D88, A:P89, A:K90, A:K91, A:R98, A:R106, A:E107, A:L108, A:I109, A:L110, A:Y111, A:D112, A:K113, A:E114, A:E115, A:R117, A:R118, A:I119, A:Q122, A:S310, A:Q311	46	0.69
1	NP	A:Q45, A:P46, A:E47, A:P48, A:C49, A:N50	6	0.947
2		A:M1, A:N2, A:P3, A:N4, A:Q5, A:K6, A:I7, A:T8, A:T9, A:I10, A:G11, A:S12, A:I13, A:C14, A:M15, A:V16, A:I17, A:G18, A:I19, A:V20, A:S21, A:L22, A:M23, A:L24, A:Q25, A:I26, A:G27, A:N28, A:I29, A:I30, A:S31, A:I32, A:W33, A:V34, A:S35, A:H36, A:S37, A:I38, A:Q39, A:T40, A:G41, A:N42, A:Q43	43	0.93
3		A:E57, A:N58, A:N59, A:T60	4	0.894
4		A:Q51, A:S52, A:I53, A:I54, A:T55, A:Y56	6	0.878
5		A:V62, A:N63, A:Q64, A:T65, A:Y66, A:V67, A:N68, A:I69, A:S70, A:N71, A:T72, A:N73	12	0.764
6		A:L140, A:N141, A:D142, A:K143	4	0.723
7		A:I108, A:G109, A:S110, A:K111, A:G112	5	0.664
8		A:G105, A:H144, A:S145, A:N146, A:G147, A:T148, A:V149, A:K150, A:I427, A:G429, A:R430, A:P431, A:K432, A:E433, A:N434, A:T435, A:I436, A:T438, A:D459, A:G460, A:A461, A:L463, A:P464, A:F465, A:T466, A:I467, A:D468	27	0.624
1	M2	A:S2, A:L3, A:L4, A:T5, A:E6, A:V7, A:E8, A:T9, A:P10, A:T11, A:K12, A:N13, A:E14, A:E16, A:N18	15	0.804
2		A:A83, A:V84, A:D85, A:V86, A:D87, A:D88, A:G89, A:H90, A:F91, A:V92, A:N93, A:I94, A:E95	13	0.774
3		A:G61, A:G62, A:P63, A:S64, A:T65, A:E66	6	0.574
4		A:S20, A:D21, A:S22, A:S23, A:D24, A:P25, A:L26, A:A29, A:A30, A:I33	10	0.556

**Table 3:** List of the predicted MHC class I epitopes of the H5N1 clade 2.3.4.4b proteins (HA, NA, NP, and M2) and their relevant information (IC50 value, percentile ranks, and allele specification).

MHC Class I Molecules						
Protein	Allele	Chicken Allele	Peptide	IC50 < 50 nM	Per Rank %	Antigenicity Score
HA	HLA-A * 11:01	BF2 * 2101	STLNQRLAPK	7.41	0.02	1.1473
	HLA-A * 02:03		RLKREEISGV	7.72	0.09	0.9344
	HLA-A * 68:01		NTQFEAVGR	10.06	0.08	1.2894
	HLA-B * 40:01		REEISGVKL	14.22	0.04	0.6846
	HLA-A * 02:03		YIVERANPA	14.9	0.24	0.7800
	HLA-A * 68:01		MNTQFEAVGR	16.2	0.16	1.1615
	HLA-B * 15:01		GQRGINSSM	22.36	0.07	1.0202
	HLA-A * 03:01		TLNQRLAPK	30.75	0.08	1.1779
	HLA-A * 30:01		KVRLQLRDNA	36.27	0.17	1.5926
	HLA-A * 68:01		MNTQFEAVGR	16.2	0.16	1.1615
	HLA-B * 15:01		GQRGINSSM	22.36	0.07	1.0202
	HLA-A * 03:01		TLNQRLAPK	30.75	0.08	1.1779
NP	HLA-C * 16:01		ATYQRTRAL	14.58	0.04	0.5864
	HLA-A * 33:01		DLRVSSFIR	38.08	0.06	0.7704
	HLA-A * 02:06		FQGRGVFEL	8.03	0.06	1.2783
	HLA-A * 11:01		GVFELTDEK	36.27	0.17	1.1503
	HLA-C * 12:03		IAYERMCNI	9.03	0.03	0.9843
	HLA-B * 07:02		KDPKKTGGPI	21.15	0.07	0.6982
	HLA-A * 68:02		NATEIRASV	17.19	0.13	0.4532
	HLA-A * 68:01		NLNDATYQR	25.72	0.28	0.6676
	HLA-A * 30:01		RTRALVRTGM	14.07	0.05	0.5749
	HLA-A * 30:01		STERATIMAA	14.96	0.06	0.4494
	HLA-A * 68:01		VASGYDFER	32.46	0.35	0.8489
NA	HLA-A * 11:01		CYPDAGDIM	15.29	0.09	0.4201
	HLA-A * 68:01		FISCSHLECR	30.11	0.4	1.0798
M2	HLA-B * 44:02		VETPTKNEW	108.42	0.1	0.6266
	HLA-A * 30:01		VYRRLKYGLK	77.63	0.39	1.2596

* Note: All listed predicted epitopes are to be probable antigenic, non-allergenic, non-toxic, and soluble.

**Table 4:** List of the predicted MHC class II of binding epitopes within different structural (HA, NP, NA, and M2) proteins of H5N1 clade 2.3.4.4b showing their IC50 value, percentile rank, and allele-specific interactions.

MHC Class II Molecules						
Protein	Allele	Chicken Allele	Peptide	IC50 < 50 nM	Per Rank %	Antigenicity Score
HA	HLA-DRB1 * 01:01	* Gaga_BLB1 * Gaga_BLB2	RVPEWSYIVERANPA	10.08	2.1	0.7022
	HLA-DRB1 * 13:02		WLIKKNDAYPTIKIS	13.85	0.46	0.9804
	HLA-DRB5 * 01:01		ATYQRTRALVRTGMD	10.99	0.15	0.4153
	HLA-DRB1 * 01:01		AELLVLMENERTLDF	15.51	4.2	1.0504
	HLA-DRB1 * 01:01		ELLVLMENERTLDFH	19.4	5.8	1.0452
	HLA-DRB1 * 13:02		LIKKNDAYPTIKISY	21.09	0.99	1.0760
	HLA-DRB1 * 13:02		RNVVWLIKKNDAYPT	25.07	1.3	1.2023
	HLA-DRB1 * 13:02		TIKISYNNTNREDLL	33.13	2.1	0.7852
	HLA-DRB1 * 04:01		PEWSYIVERANPAND	33.88	0.55	0.7539
	HLA-DRB1 * 11:01		FRNVVWLIKKNDAYP	37.72	2	1.1509
	HLA-DRB1 * 13:02		AYPTIKISYNNTNRE	38.33	2.5	0.8365
	HLA-DRB1 * 13:02		PTIKISYNNTNREDL	41.05	2.8	0.7790
NP	HLA-DRB1 * 11:01		MELIRMIKRGINDRN	9.21	0.14	0.5862
	HLA-DRB1 * 07:01		AEIEDLIFLARSALI	10.77	0.29	0.8823
	HLA-DRB5 * 01:01		ATYQRTRALVRTGMD	10.99	0.15	0.4153
	HLA-DRB1 * 15:01		EDLIFLARSALILRG	14.06	0.17	0.7376
	HLA-DRB1 * 07:01		EIEDLIFLARSALIL	14.83	0.74	0.9266
	HLA-DRB1 * 01:01		PRMCSLMQGSTLPRR	15.32	4.1	0.4574
	HLA-DRB5 * 01:01		DATYQRTRALVRTGM	15.37	0.53	0.5614
	HLA-DRB1 * 01:01		RMCSLMQGSTLPRRS	16.83	4.8	0.5336
	HLA-DRB5 * 01:01		GRFYIQMCTELKLSD	17.36	0.64	0.4565
	HLA-DRB1 * 01:01		DPRMCSLMQGSTLPR	20.4	6.1	0.4614
	HLA-DQA1 * 05:01/DQB1 * 03:01		PRRSGAAGAAVKGVG	28.48	1.2	0.9345
	HLA-DQA1 * 05:01/DQB1 * 03:01		LPRRSGAAGAAVKGV	29.2	1.2	0.8733
	HLA-DRB5 * 01:01		SSFIRGTRVVPRGQL	30.02	1.8	0.5929
	HLA-DQA1 * 04:01/DQB1 * 04:02		ARSALILRGSVAHKS	41.48	0.49	0.6766
	HLA-DRB5 * 01:01		RSALILRGSVAHKSC	41.78	2.9	0.6269
	HLA-DQA1 * 05:01/DQB1 * 03:01		TLPRRSGAAGAAVKG	42.77	2.3	0.8370
	HLA-DQA1 * 04:01/DQB1 * 04:02		RSALILRGSVAHKSC	45.19	0.7	0.6269
	HLA-DPA1 * 03:01/DPB1 * 04:02		GRRTRIAYERMCNIL	46.18	0.71	0.6312
	HLA-DRB5 * 01:01		VGTMVMELIRMIKRG	48.56	3.6	0.4815
	HLA-DPA1 * 01:03/DPB1 * 02:01		FEDLRVSSFIRGTRV	49.13	1.4	0.8472
	HLA-DQA1 * 05:01/DQB1 * 03:01		LPRRSGAAGAAVKGV	29.2	1.2	0.8733
	HLA-DRB5 * 01:01		SSFIRGTRVVPRGQL	30.02	1.8	0.5929
NA	HLA-DRB3 * 01:01		WAIYSKDNGIRIGSK	16.43	0.21	0.9819
	HLA-DRB1 * 01:01		SFKYGNGVWIGRTKS	25.69	7.9	1.2583
M2	HLA-DRB1 * 11:01		VETPTKNEW	108.42	0.1	0.6266
	HLA-DRB1 * 01:01		VYRRLKYGLK	77.63	0.39	1.2596
	HLA-DRB5 * 01:01		DRLFFKCVYRRLKYG	23.64	0.92	0.4858
	HLA-DPA1 * 01:03/DPB1 * 02:01		SFKYGNGVWIGRTKS	25.69	7.9	1.2583
	HLA-DRB5 * 01:01		CVYRRLKYGLKGGPS	103.77	8.3	1.1811
	HLA-DRB3 * 01:01		DRLFFKCVYRRLKYG	115.81	3.8	0.4858
	HLA-DRB3 * 01:01		KCVYRRLKYGLKGGP	76.56	6.1	0.9916
	HLA-DRB5 * 01:01		QQSAVDVDDGHFVNI	113.4	2.6	1.0804
	HLA-DRB1 * 11:01		QSAVDVDDGHFVNIE	134.91	3	1.1815

* Note: All listed predicted epitopes are to be probable antigenic, non-allergenic, non-toxic, and soluble.

**Table 5:** The sequences and the relevant information of the top-ranked selected epitopes used for the construction of the multiepitope DNA-based vaccine against H5N1 clade 2.3.4.4b.

S.No	Protein	Start	Peptide	Antigenicity Score	Docking Score	Confidence Score (>0.8)
MHC class I molecules						
1	HA	406	KVRLQLRDNA	1.5926	−188.17	0.6821
2	NP	398	FQGRGVFEL	1.2783	−214.75	0.7850
3	NA	121	FISCSHLECR	1.0798	−214.75	0.7850
4	M2	51	VYRRLKYGLK	1.2596	−178.50	0.6388
MHC class II molecules						
1	HA	41	RNVVWLIKKNDAYPT	1.2023	−263.89	0.9070
2	NP	252	EIEDLIFLARSALIL	0.9266	−214.79	0.7851
3	NA	350	SFKYGNGVWIGRTKS	1.2583	−255.61	0.8921
4	M2	51	VYRRLKYGLKGGPST	1.2088	−249.52	0.8798
B cell epitopes						
1	HA	168	KKNDAYPTIKISYNNTNRED	1.1073		
2	NP	200	MSSNGAYGVKGFSFKYGNGV	0.9688		
3	NA	338	GINDRNFWRGENGRRTRIAY	0.9417		
4	M2	56	KYGLKGGPSTEGVPESMREE	0.8569		

* Note: All listed predicted epitopes are to be probable antigenic.

## Data Availability

The raw data supporting the conclusions of this article will be made available by the authors upon request.

## Supplementary Materials

The following supporting information can be downloaded at https://www.mdpi.com/article/10.3390/v17091152/s1, Table S1: HA of 279 isolates of H5N1 clade 2.3.4.4b; Table S2: NA of 79 isolates of H5N1 2.3.4.4b Chicken; Table S3: NP of 79 isolates of H5N1 2.3.4.4b; Table S4: M2 of 79 isolates of H5N1 2.3.4.4b; Figure S1: Phylogenetic tree of HA protein derived from 279 H5N1 clade 2.3.4.4b isolates. The phylogenetic tree was constructed based on the neighbor-joining method using Geneious Prime software (https://www.geneious.com/ (accessed on 1 August 2025)). The resulting Nexus file was used to develop a phylogenetic tree figure using online tool iTOL (https://itol.embl.de/ (accessed on 1 August 2025)); Figure S2: Phylogenetic tree of M2 protein derived from 279 H5N1 clade 2.3.4.4b isolates. The phylogenetic tree was constructed based on the neighbor-joining method using Geneious Prime software (https://www.geneious.com/ (accessed on 1 August 2025)). The resulting Nexus file was used to develop a phylogenetic tree figure using the online tool iTOL (https://itol.embl.de/ (accessed on 1 August 2025)); Figure S3: Phylogenetic tree of NA protein derived from 279 H5N1 clade 2.3.4.4b isolates. The phylogenetic tree was constructed based on the neighbor-joining method using Geneious Prime software (https://www.geneious.com/ (accessed on 1 August 2025)). The resulting Nexus file was used to develop a phylogenetic tree figure using online tool iTOL (https://itol.embl.de/ (accessed on 1 August 2025); Figure S4: Phylogenetic tree of NP protein derived from 279 H5N1 clade 2.3.4.4b isolates. The phylogenetic tree was constructed using the neighbor-joining method in Geneious Prime software (https://www.geneious.com/ (accessed on 1 August 2025)). The resulting Nexus file was used to develop a phylogenetic tree figure using online tool iTOL (https://itol.embl.de/ (accessed on 1 August 2025)); Figure S5: Molecular docking analysis of multiepitope vaccine construct of H5N1 clade 2.3.4.4b with the chicken Toll-like immune receptor (TLR7) using Biovia Discovery Studio, (A) Topology illustration of the chicken TLR3 protein structure analyzed by the PDBsum server, (B) The docking results of the binding interaction between the designed vaccine construct and the TLR7, (C) the interaction residues; (D) and its binding amino acids between vaccine construct and TLR7 using PDBsum server; Figure S6: The vector map shows in silico cloning of the multiepitope H5N1 clade 2.3.4.4b (HA, NP, NA, and M2) into the pET-28 (+) Expression Vector; Figure S7: In silico immune simulation analysis of the H5N1 clade 2.3.4.4b multiepitope vaccine construct (A) The kinetics profile displaying antigen-immunoglobulin production (IgM, IgG1, IgG2, and IgM + IgG), (B) B lymphocytes population per entity-state (i.e., showing counts for active, presenting on class-II, internalized the Ag, duplicating and anergic, (C) B lymphocytes total count, memory cells, and sub-divided in isotypes IgM, IgG1 and IgG2, (D) CD4 T-helper lymphocytes count sub-divided per entity-state (i.e., active, resting, anergic and duplicating), (E) CD4 T-helper lymphocytes count. The plot shows total and memory counts, as well as (F) CD4 T-regulatory lymphocyte count. Both total memory and per-entity-state counts are plotted: (G) The CD8 T-cytotoxic lymphocytes count per entity-state, and (H) the CD8 T-cytotoxic lymphocytes count. Total and memory shown, (I) Dendritic cells. The DC can present antigenic peptides on both MHC class-I and class-II molecules, (J) epithelial cells. The total count is broken down into active, virus-infected, and presented on class-I MHC molecule, (K) Macrophages. Total count, internalized, presenting on MHC class-II, active and resting macrophages, (L) Natural Killer cells (total count), (M) Plasma B lymphocyte count subdivided per isotype (IgM, IgG1, and IgG2). The simulation was performed utilizing the antigen-combined sequence data from four proteins (HA, NA, NP, and M2) of the H5N1 clade 2.3.4.4b, and it was set to 100 with a volume of 10.

## Abbreviations

HA	Hemagglutinin
NA	Neuraminidase
NP	Nucleoprotein
M	Matrix protein
HPAI	Highly pathogenic avian influenza
ML	Machine learning
AI	Artificial intelligence
IL8	Interleukin-8
NA	Not applicable
mAbs	Monoclonal antibodies
RMSD	Root mean square deviation
