# Supplementary material for: A Pan-H5N1 Multiepitope DNA Vaccine Construct Targeting Some Key Proteins of the Clade 2.3.4.4b Using AI-Assisted Epitope Mapping and Molecular Docking
Source: Viruses. 2025 Aug 22;17(9):1152. doi: 10.3390/v17091152 (PMC12474345; doi:10.3390/v17091152)
Supplement: Supplementary file 1 [file viruses-17-01152-s001.zip › Supplementary Figures S1-S4.pdf]

Supplementary Figures:

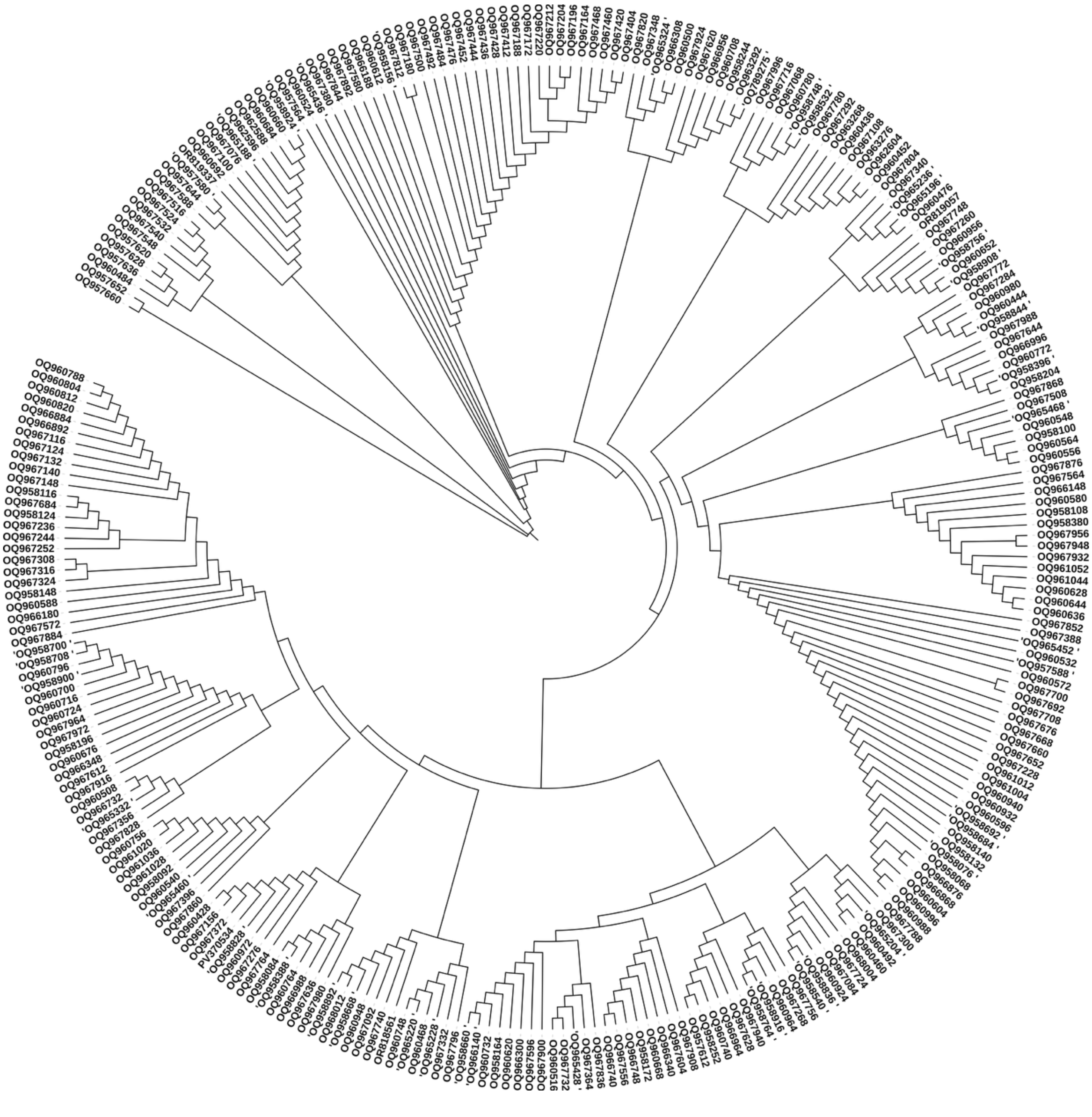

Supplementary Figure S1: Phylogenetic tree of HA protein derived from 279 H5N1 clade 2.3.4.4b isolates.

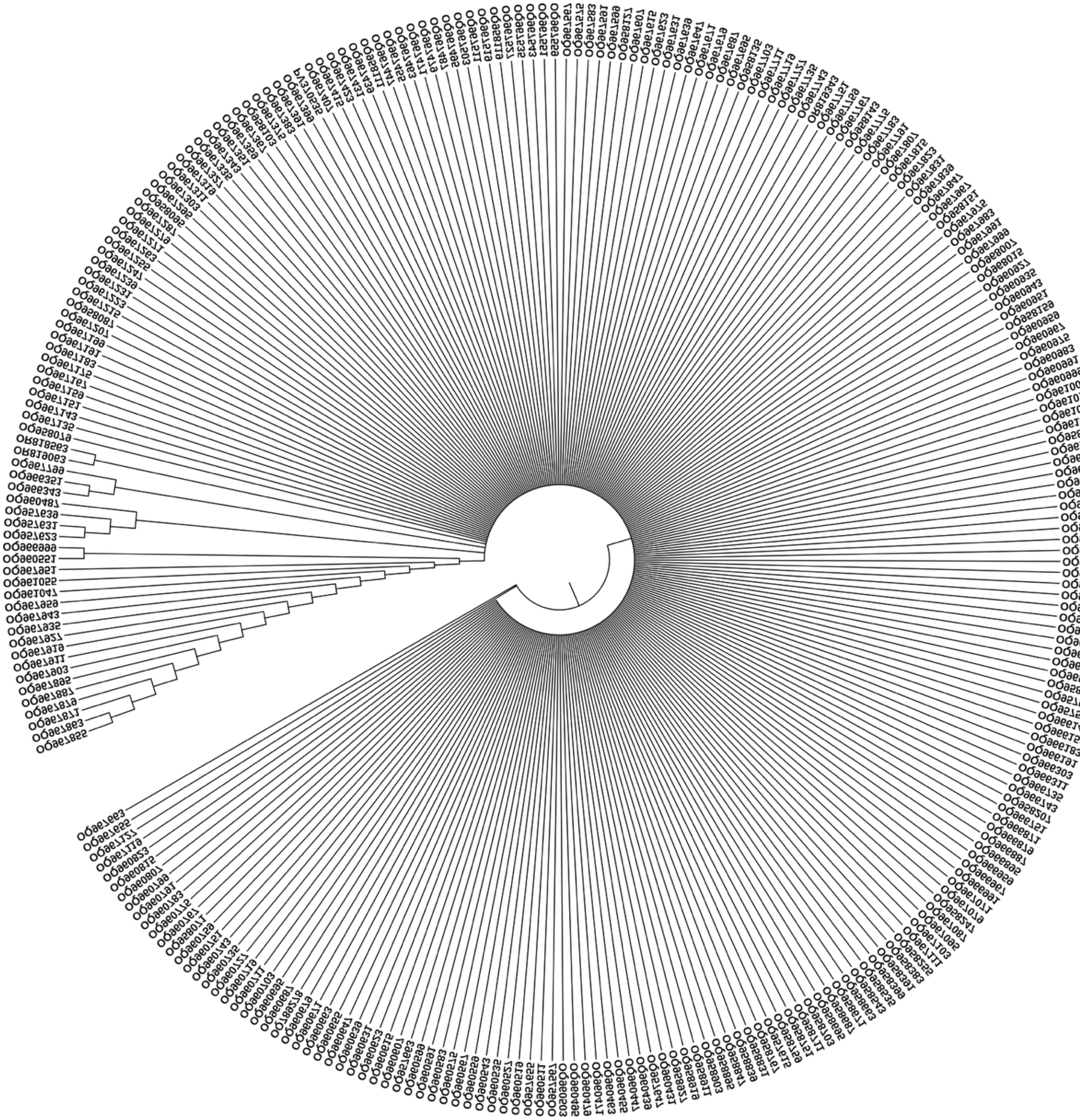

Supplementary Figure S2: Phylogenetic tree of M2 protein derived from 279 H5N1 clade 2.3.4.4b isolates.

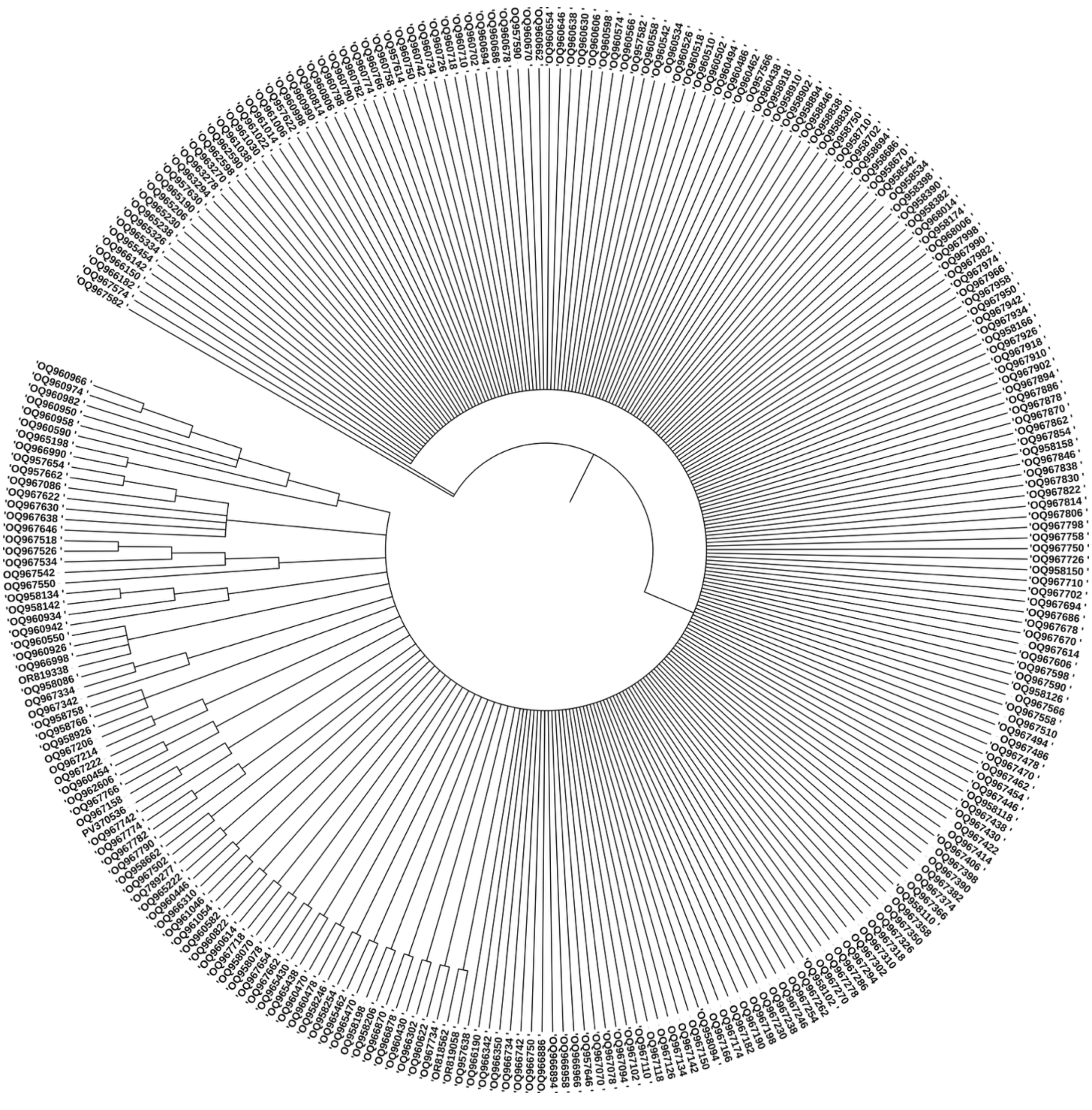

Supplementary Figure S3: Phylogenetic tree of NA protein derived from 279 H5N1 clade 2.3.4.4b isolates.

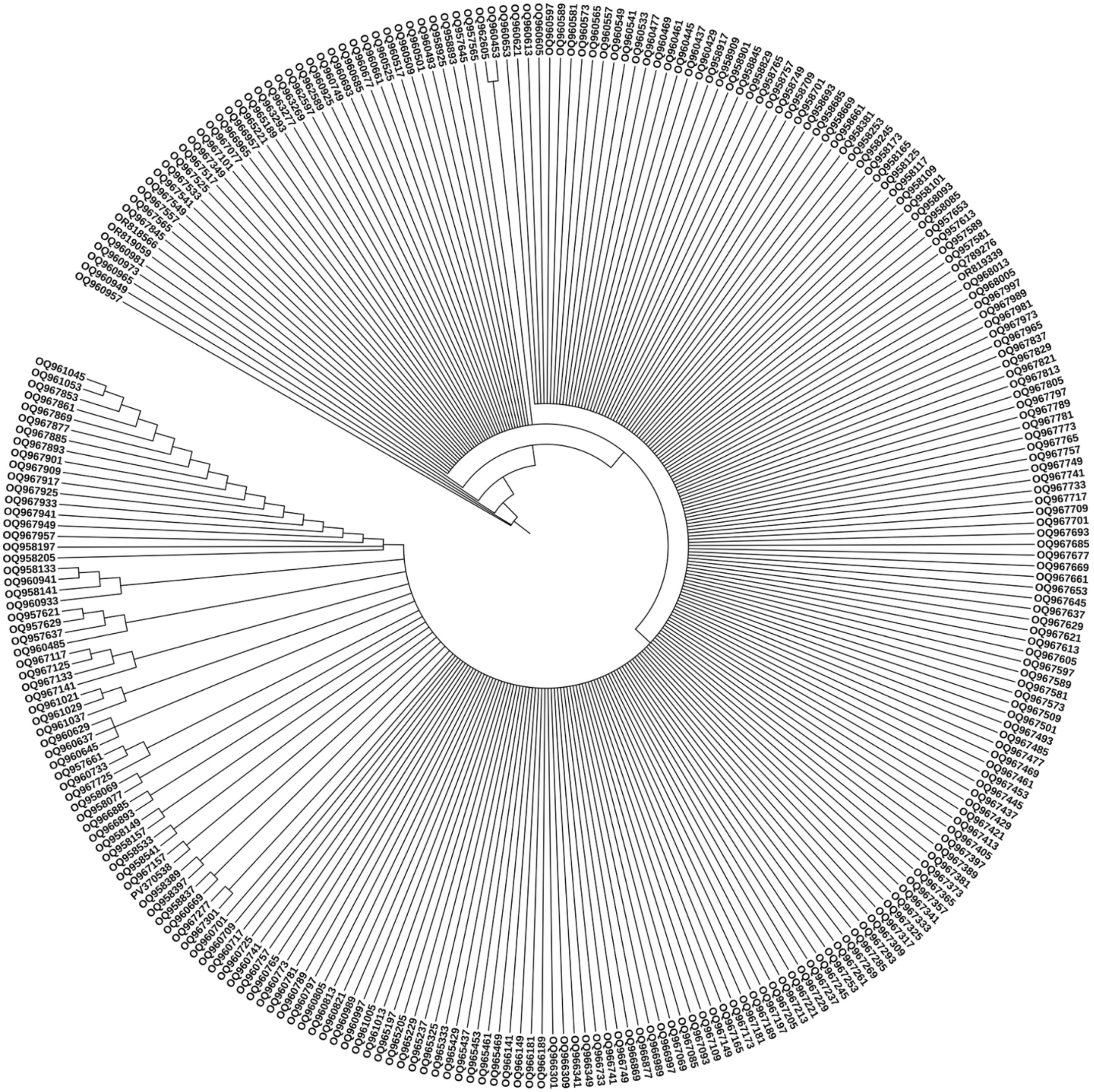

Supplementary Figure S4: Phylogenetic tree of NP protein derived from 279 H5N1 clade 2.3.4.4b isolates.

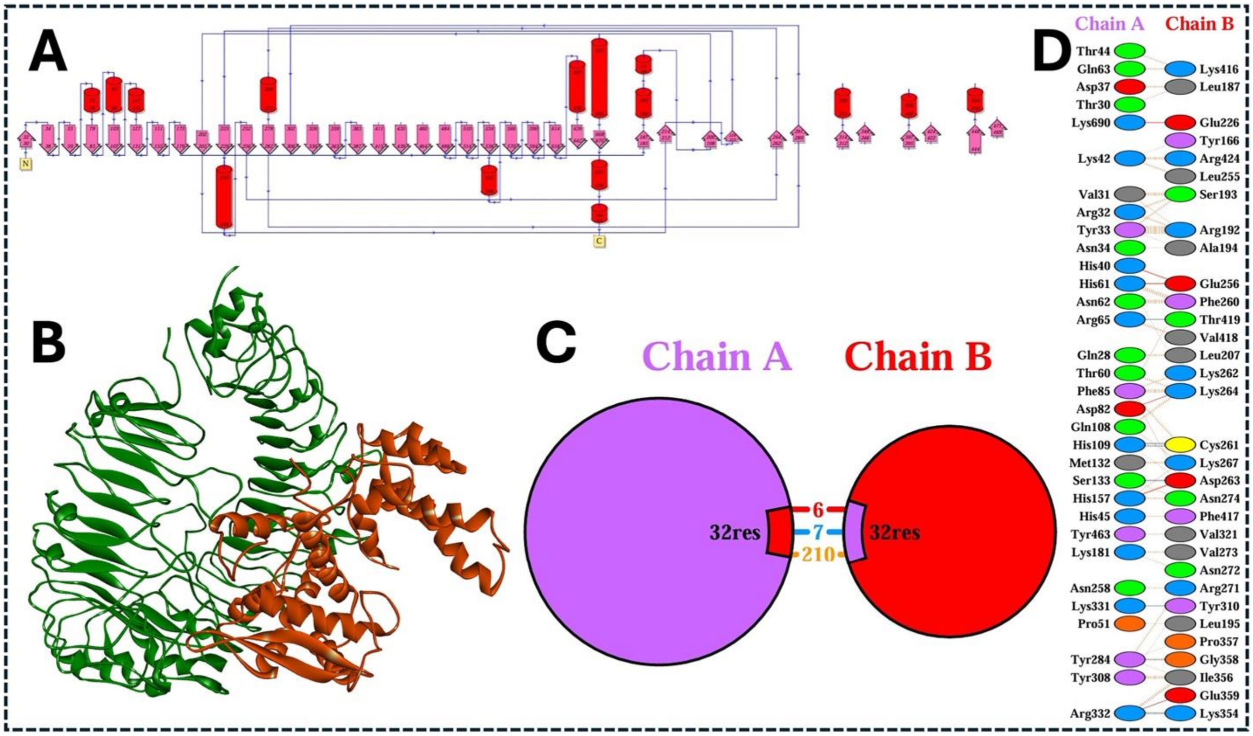

Supplementary Figure S5: Molecular docking analysis of multiepitope vaccine construct of H5N1 clade 2.3.4.4b with the chicken Toll-like immune receptor (TLR7).

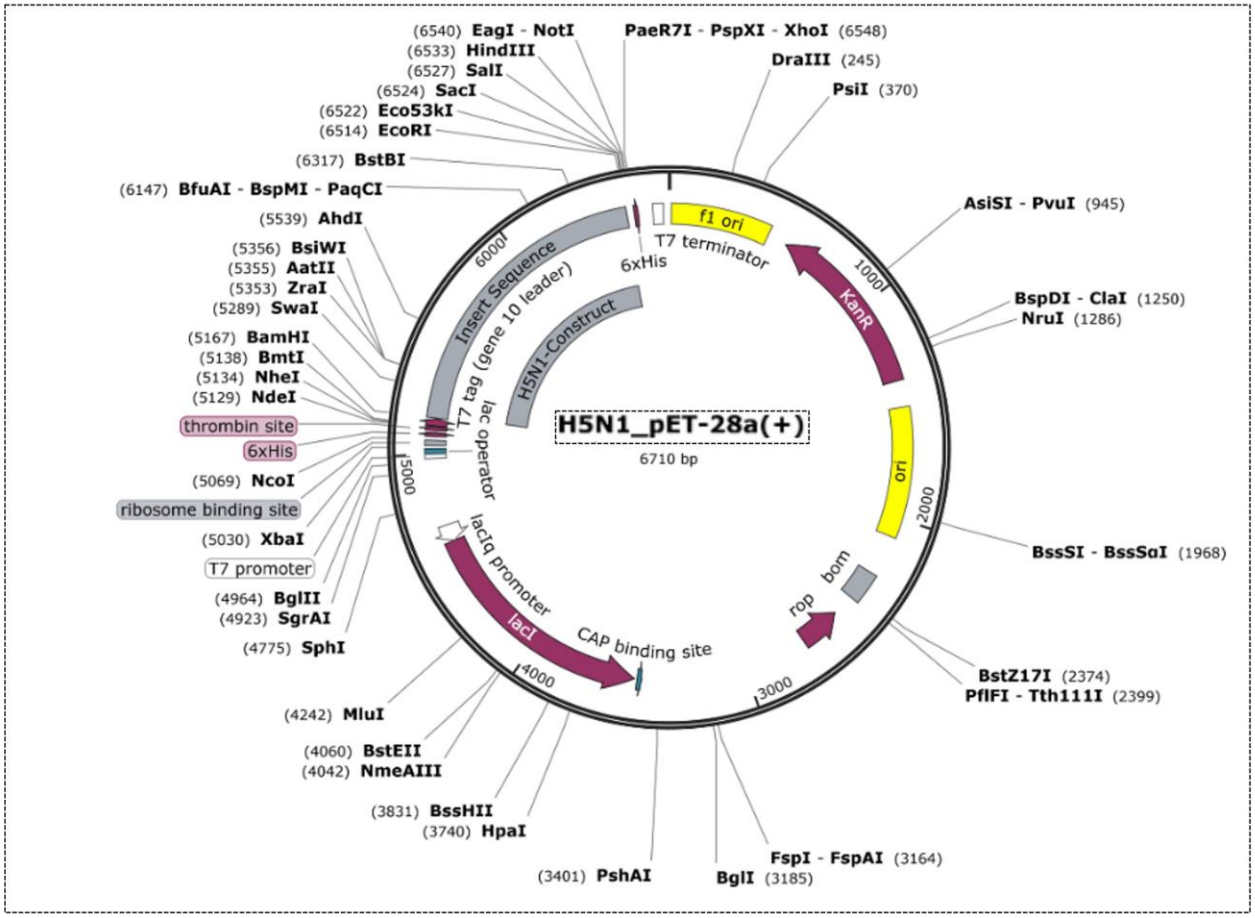

Supplementary Figure S6: The vector map shows in silico cloning of the multiepitope H5N1 clade 2.3.4.4b (HA, NP, NA, and M2) into the pET-28(+) Expression Vector.

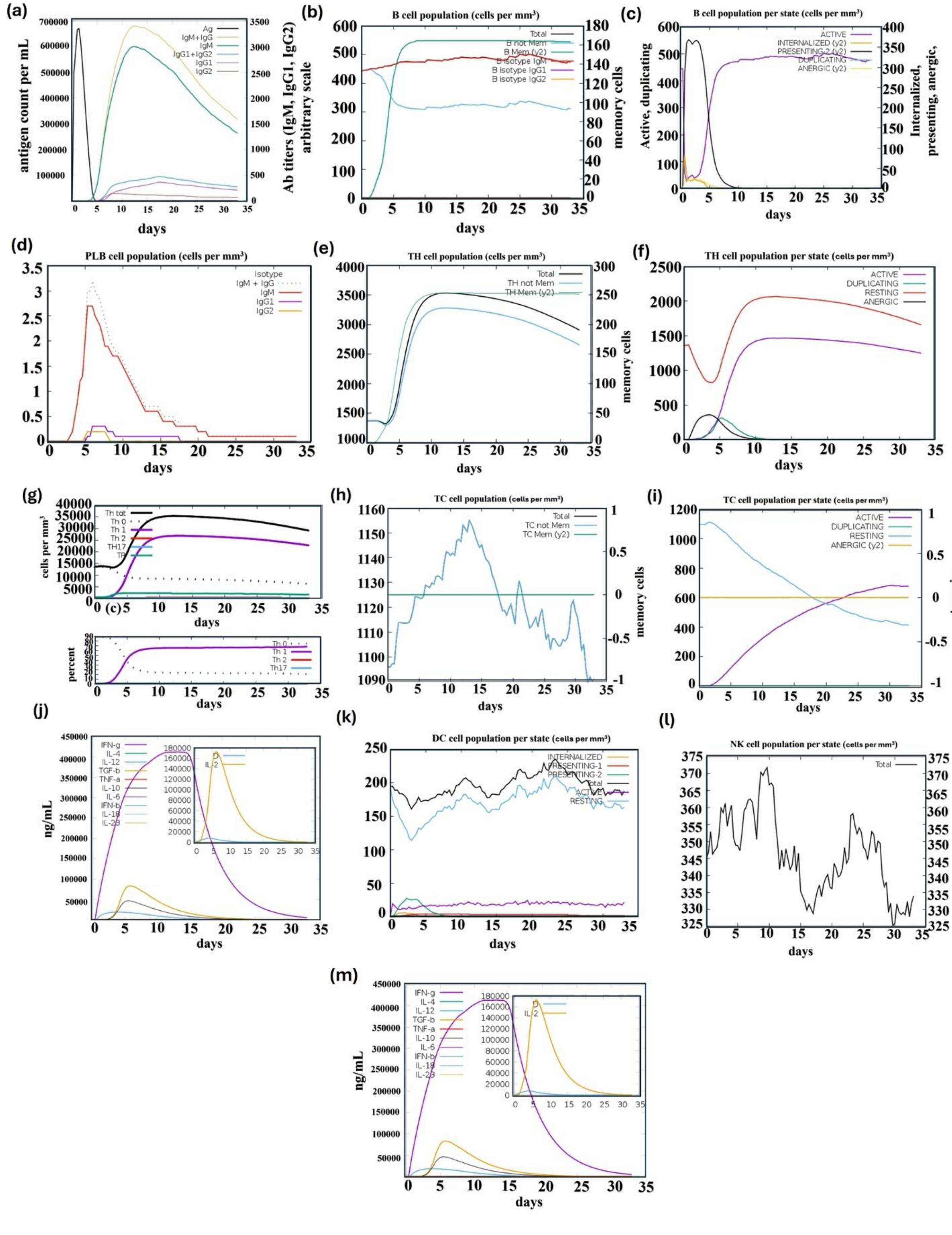

Supplementary Figure S7: In silico immune simulation analysis of the H5N1 clade 2.3.4.4b multiepitope vaccine construct
