# Supplementary figures and images for: A Pan-H5N1 Multiepitope DNA Vaccine Construct Targeting Some Key Proteins of the Clade 2.3.4.4b Using AI-Assisted Epitope Mapping and Molecular Docking
Source: Viruses. 2025 Aug 22;17(9):1152. doi: 10.3390/v17091152 (PMC12474345; doi:10.3390/v17091152)

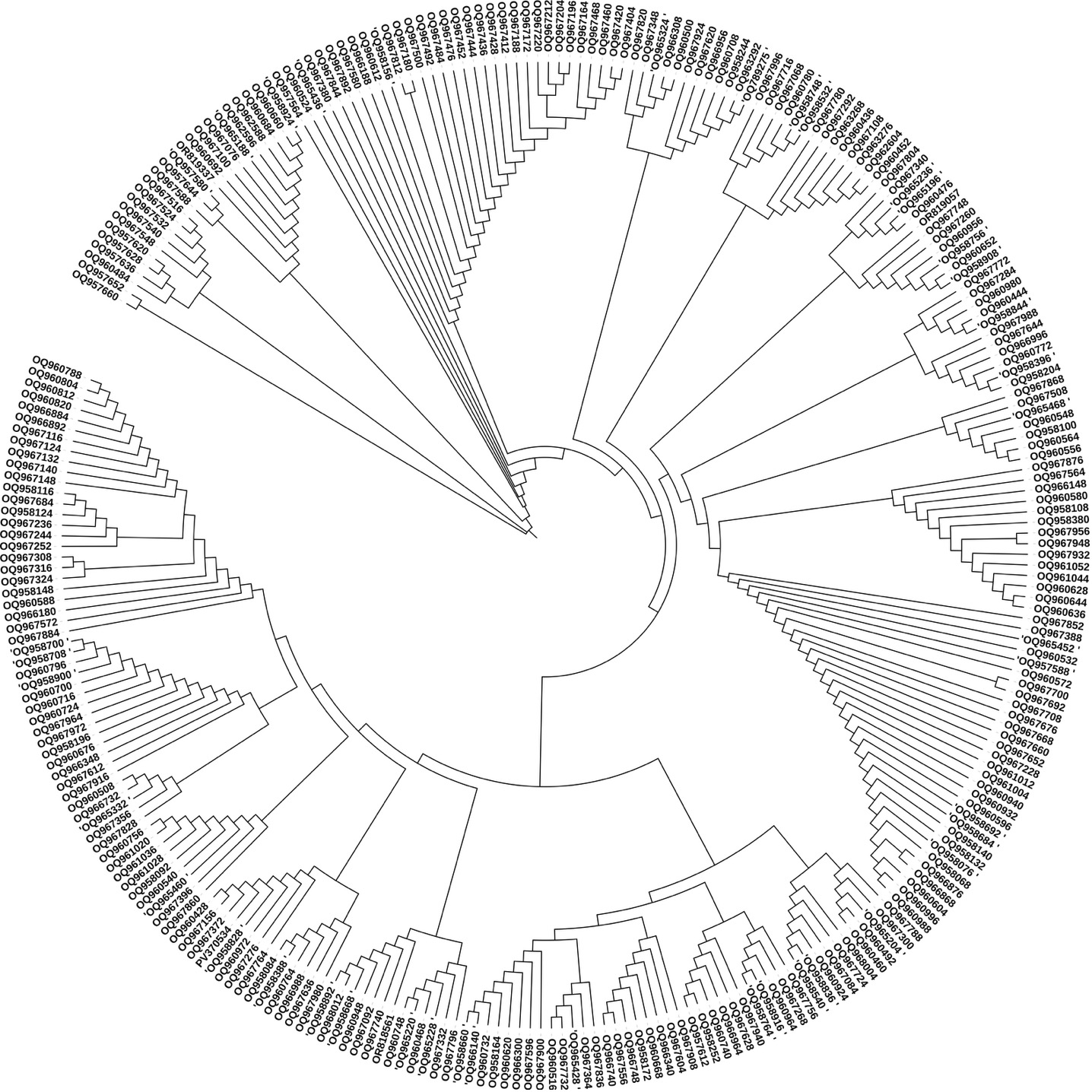

Supplement: Supplementary file 1 [file viruses-17-01152-s001.zip › Supp Figures/Supplementary Figure S1.jpg]

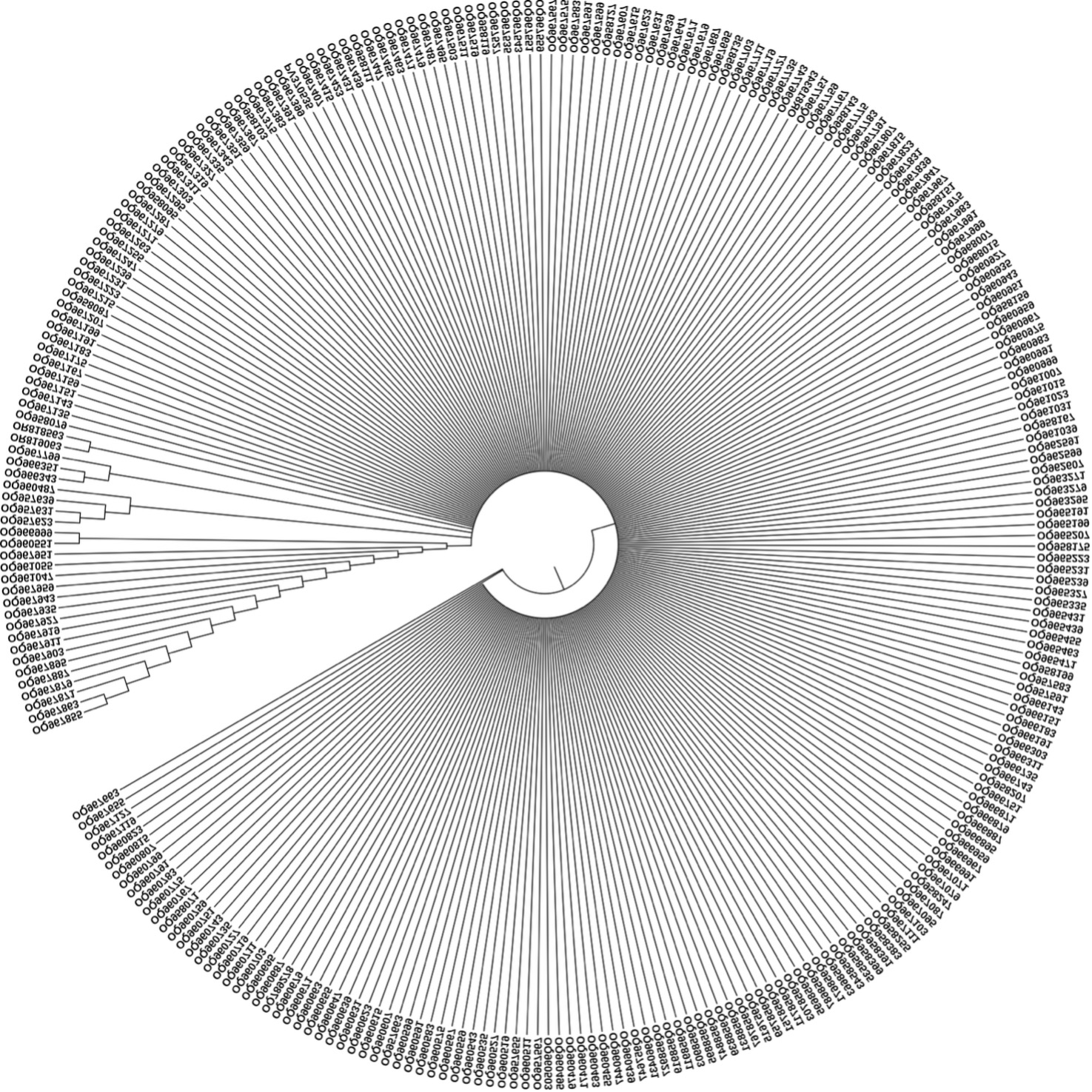

Supplement: Supplementary file 1 [file viruses-17-01152-s001.zip › Supp Figures/Supplementary Figure S2.jpg]

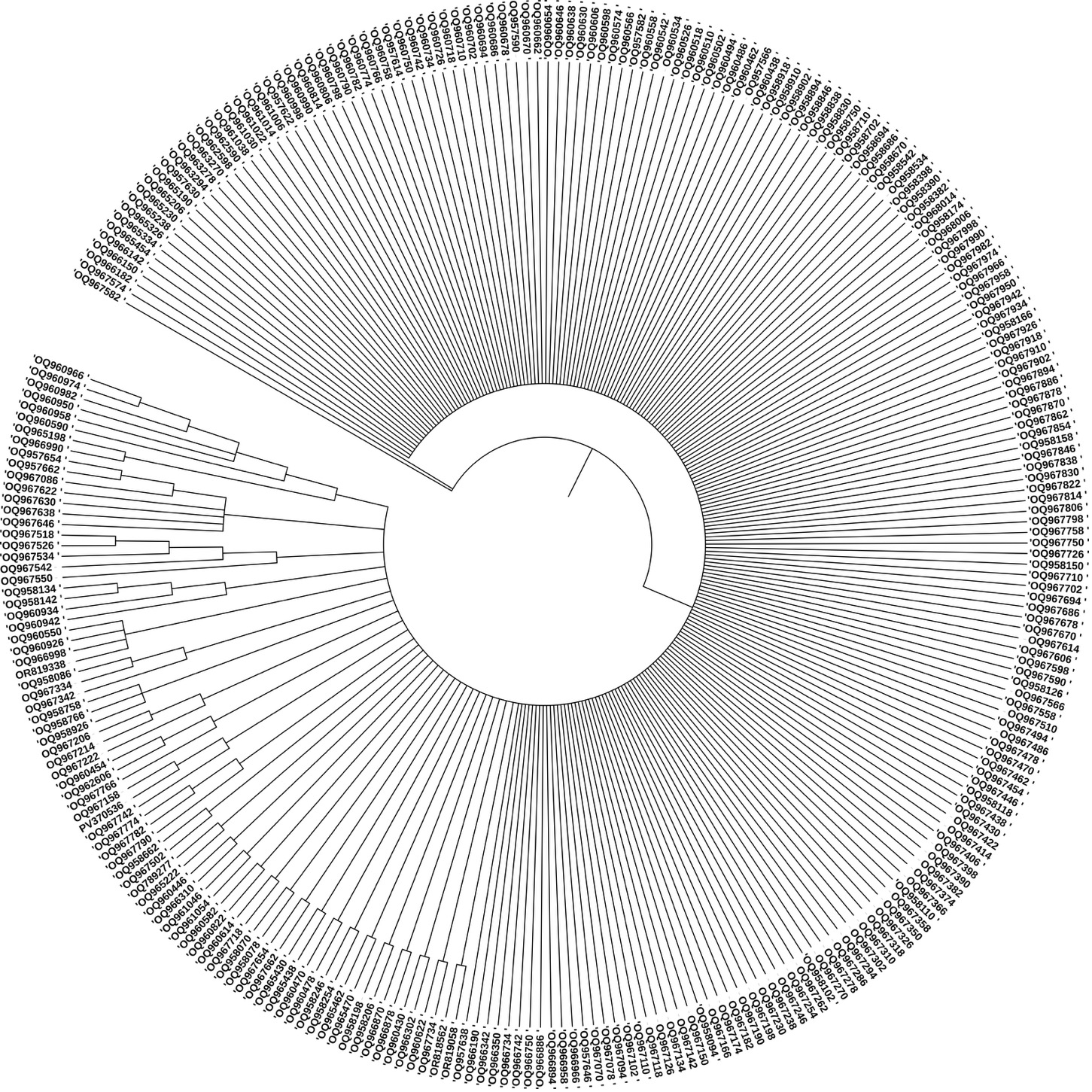

Supplement: Supplementary file 1 [file viruses-17-01152-s001.zip › Supp Figures/Supplementary Figure S3.jpg]

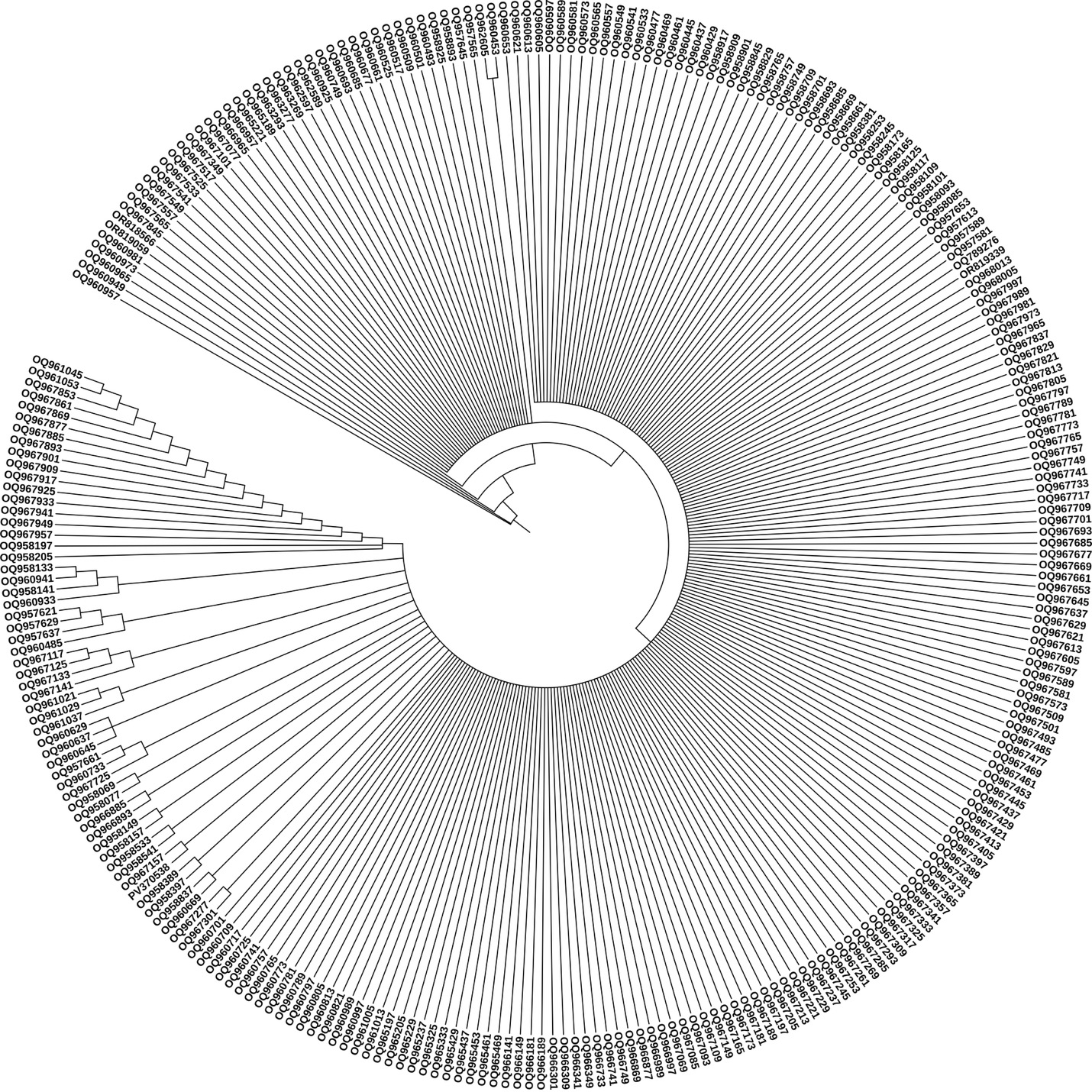

Supplement: Supplementary file 1 [file viruses-17-01152-s001.zip › Supp Figures/Supplementary Figure S4.jpg]

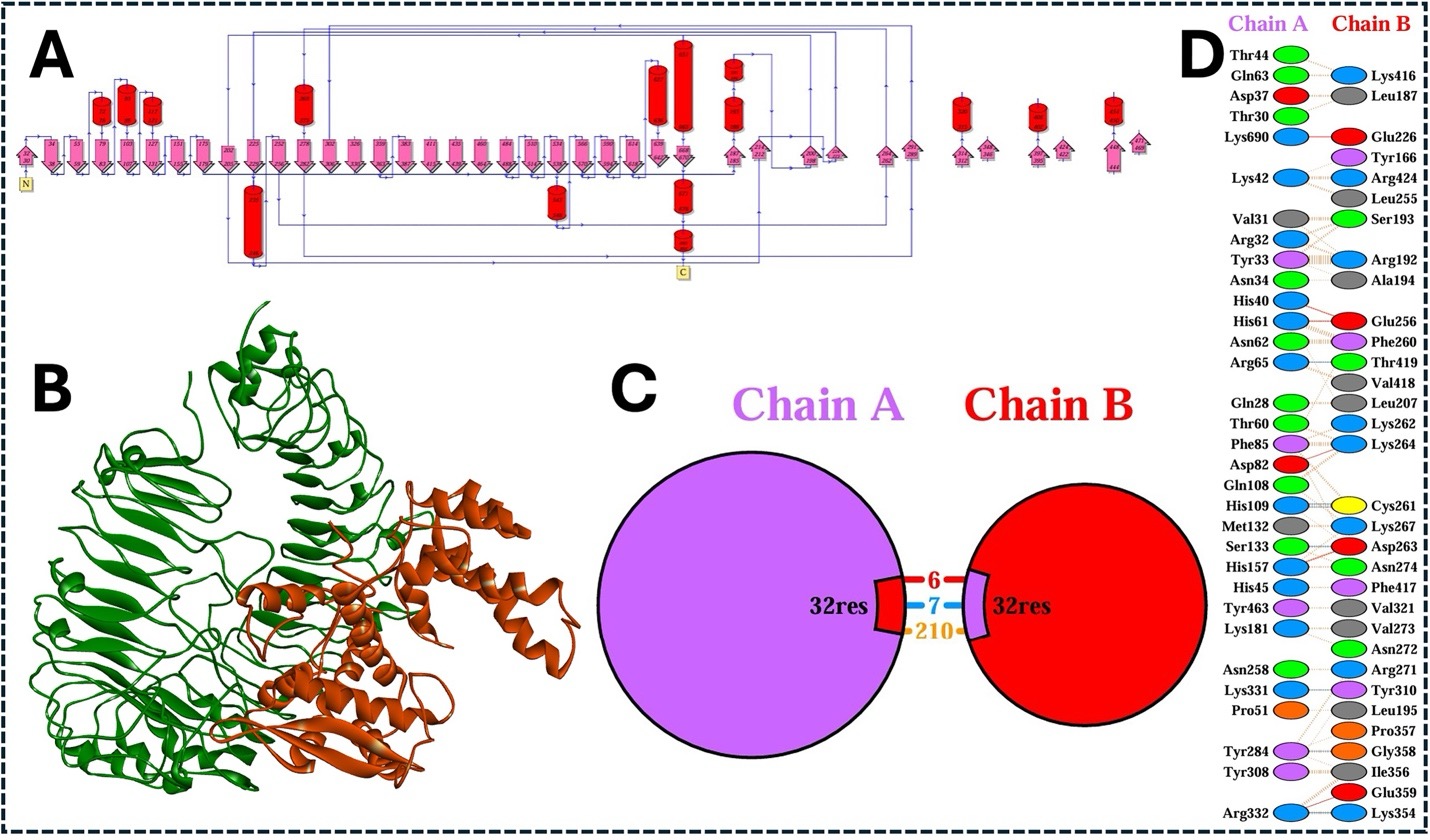

Supplement: Supplementary file 1 [file viruses-17-01152-s001.zip › Supp Figures/Supplementary Figure S5.jpg]

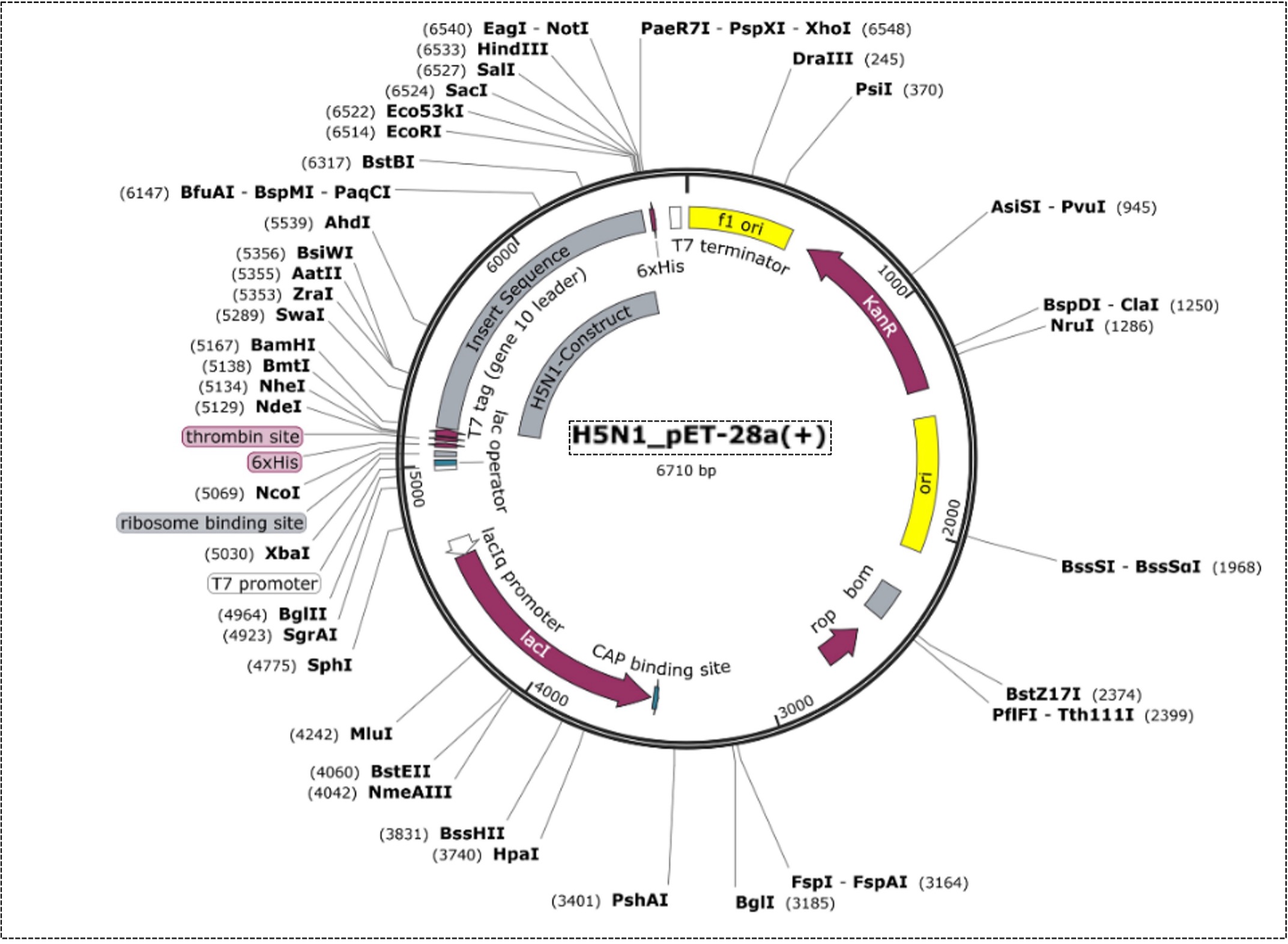

Supplement: Supplementary file 1 [file viruses-17-01152-s001.zip › Supp Figures/Supplementary Figure S6.jpg]

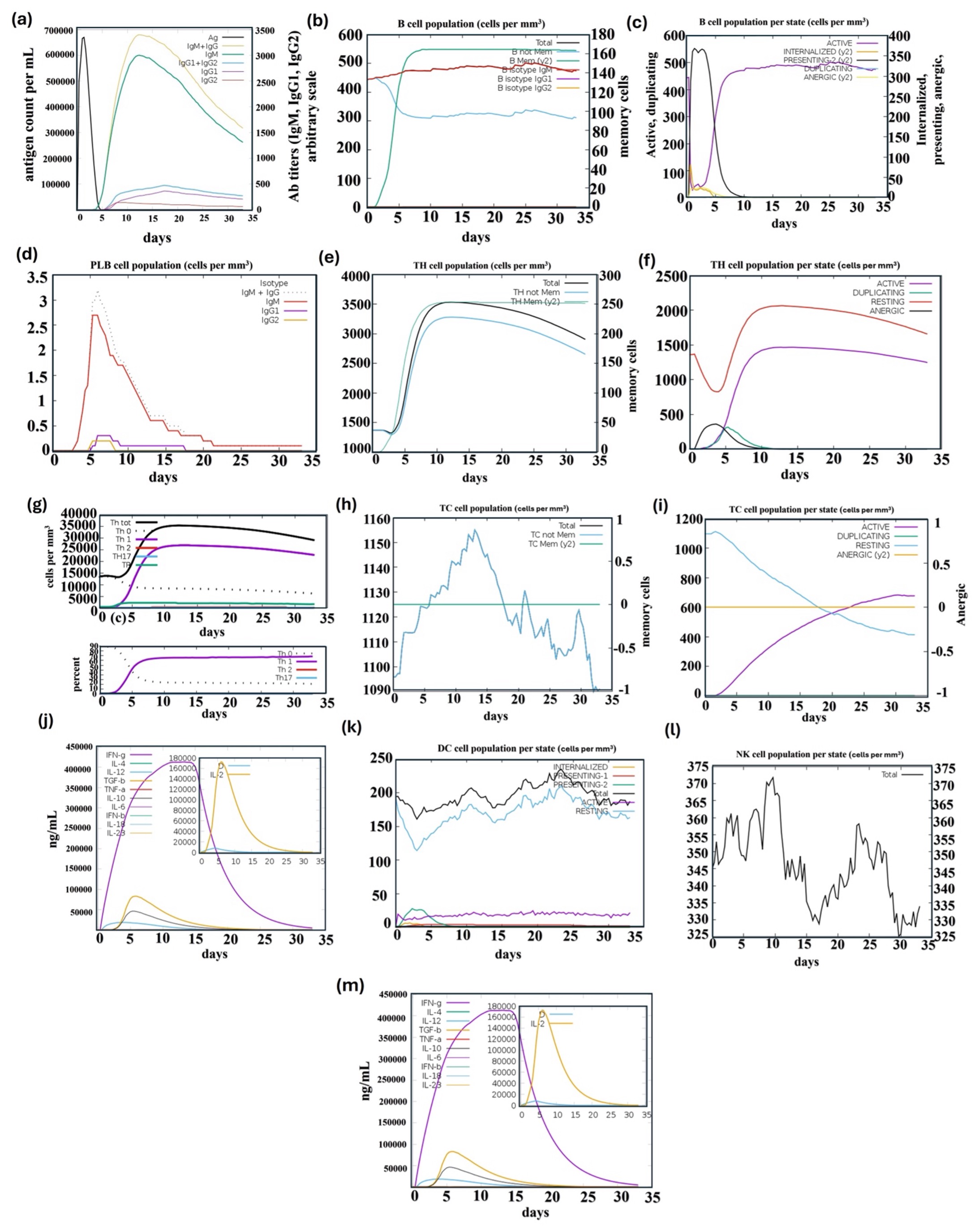

Supplement: Supplementary file 1 [file viruses-17-01152-s001.zip › Supp Figures/Supplementary Figure S7.jpg]
